# Predictors of increasing disability in activities of daily living among people with advanced respiratory disease: a multi-site prospective cohort study, England UK

**DOI:** 10.1080/09638288.2023.2288673

**Published:** 2023-12-10

**Authors:** Lucy Fettes, Joanne Bayly, Emeka Chukwusa, Stephen Ashford, Irene Higginson, Matthew Maddocks

**Affiliations:** Cicely Saunders Institute of Palliative Care, Policy and Rehabilitation, King’s College London, London, UK

**Keywords:** Activities of daily living, disability, rehabilitation, advanced cancer, respiratory disease, palliative care

## Abstract

**Purpose:**

Disability in activities of daily living (ADL) is a common unmet need among people with advanced respiratory disease. Rehabilitation could help prolong independence, but indicators for timely intervention in this population are lacking. This study aimed to identify trajectories of disability in ADLs over time, and predicting factors, in advanced respiratory disease.

**Method:**

Multi-site prospective cohort study in people with advanced non-small cell lung cancer (NSCLC), chronic obstructive pulmonary disease (COPD) or interstitial lung disease (ILD), recruited from hospital or community services, throughout England. Disability in basic (Barthel Index) and instrumental (Lawton–Brody IADL Scale) ADLs were assessed monthly over six months. Visual graphical analysis determined individual trajectories. Multivariate logistic regression examined predictors of increasing disability in basic and instrumental ADLs.

**Findings:**

Between March 2020 and January 2021, we recruited participants with a diagnosis of NSCLC (*n* = 110), COPD (*n* = 72), and ILD (*n* = 19). 151 participants completed ≥3 timepoints and were included in the longitudinal analysis. Mobility limitation was an independent predictor of increasing disability in instrumental ADLs (odds ratio, 1⋅41 [CI: 1⋅14–1⋅74], *p* = 0⋅002).

**Conclusion:**

Mobility limitation could be used as a simple referral criterion across people with advanced respiratory disease to ensure timely rehabilitation that targets independence in ADLs.

## IMPLICATION FOR REHABILITATION

- To our knowledge this is the first prospective cohort study of trajectories of disability in activities of daily living (ADL) in advanced respiratory disease, including recruitment during the Covid-19 pandemic.
- It adds to existing evidence by identifying individual variability in trajectories of ADL disability which are undetected at group level.
- The identification of mobility limitation as a predictor of increasing ADL disability, while controlling for malignant or non-malignant respiratory disease, is novel and has practical utility.
- Our findings have implications for clinical care, as early identification of functional decline through use of mobility limitation tools could flag early referral to rehabilitation services, potentially preventing or delaying forthcoming functional decline and avoiding reactive crisis management.
- Mobility limitation is a predictor of increasing disability in activities of daily living in advanced disease, which could be used to flag early referral to rehabilitation services, to help prevent or delay forthcoming functional decline and avoid reactive crisis management

## Introduction

Respiratory disease is any disease that obstructs or restricts the respiratory system, which can be malignant (e.g., lung cancer) or non-malignant (e.g., chronic obstructive pulmonary disease (COPD) or Interstitial lung disease (ILD)) [1]. In the UK alone, an estimated 85,000 people are living with lung cancer, 1.2 million people are living with chronic obstructive pulmonary disease (COPD) and 32,500 people are living with interstitial lung disease (ILD) [1]. The 2019 Global Burden of Disease Study found non-malignant and malignant respiratory disease were the third and fourth leading cause of death and productive life lost due to disability worldwide respectively [2].

Activities of daily living (ADLs) describe a collection of skills required to live independently, which can be considered either basic ADLs (e.g., washing, dressing, bathing, toileting, feeding), or as more complex instrumental ADLs (e.g., shopping, housework, use of public transportation) [3]. Disability in ADLs (ADL disability) describes a person having difficulty or becoming unable to perform ADLs independently. This disability is underpinned by a complex relationship between an individual’s health condition, impairments to body functions and structures, and contextual factors including personal attributes and the environment in which a person lives [4].

Functional decline is common in advanced respiratory disease and consequent disability in ADLs is highly prevalent [5,6]. It is almost inevitable for patients with advanced lung cancer, COPD or ILD to experience an element of difficulty managing ADLs, and over 50% of this population is likely to develop dependency in ADLs and need support to undertake everyday tasks [7]. Increased life-expectancy resulting from unprecedented population ageing at a global level [8], means people with advanced respiratory disease will potentially be living with ADL disability for longer periods [2]. In addition, the prevalence of disability in advanced respiratory disease is likely to increase as a result of the Covid-19 pandemic [9]. These factors all contribute to increase demand on health and social care services and potentially need for formal care [10].

There are drives to increase provision of specialist services targeting functional decline in advanced respiratory disease [11], given ADL disability is one of the most common unmet supportive care needs in this population [5,12]. It is important to improve understanding of disability in advanced respiratory disease in order to identify people most likely to experience functional decline and dependency in ADL [13]. This could improve timing and delivery of rehabilitation interventions to optimize function and reduce disabilities that individuals experience in interaction with their environment [14]. Ultimately this can prolong independence in ADLs and delay the need for or extent of care [15].

Evidence from pre-existing studies of trajectories of disability in activities of daily living [8, 10], are limited by use of retrospective data from the point of death, samples of community dwelling elders rather than a specific sample of advanced cancer or respiratory disease, proxy reporting, invalidated measures of ADL, and infrequent timing of measurement missing possible fluctuations in the trajectory. This justifies the need for a longitudinal study of ADL disability trajectory that addresses these methodological limitations, and robustly answers the question of: *“what is the course of ADL disability in advanced respiratory disease and is it possible to identify predictors of forthcoming dependency in ADLs in this population?”.* Consequently, this study aims to describe the course of disability in ADLs over time and identify predictors of increasing disability in ADLs to inform clinical care for people with advanced respiratory disease.

## Materials and methods

### Study design

We report longitudinal data of a multi-site prospective cohort study, following the STROBE guidelines [16] (STROBE checklist in supplementary material A). The study was registered on the ISRCTN registry (ISRCTN14159936) and ethical approval was granted by the London Camberwell St Giles Research Ethics Committee (ref 19/LO/1950). The study protocol can be found in Supplementary material B.

### Participants

The study population is restricted to lung cancer and respiratory disease (COPD/ILD), which account for over 60% of all deaths from respiratory disease [1]. The sample size calculation considered that comparisons would be made between these two diagnostic groups. Including other relatively rare respiratory diseases such as Pulmonary hypertension and Cystic fibrosis, in the sample would mean there would be a risk of small groups of patients in our study, which would lead to very imprecise estimates.

Inclusion criteria were adults with a diagnosis of either: i) Inoperable stage III or IV non-small cell lung cancer (NSCLC); ii) Severe or very severe Chronic Obstructive Lung Disease (COPD), defined by FEV_1_ <50% predicted [17]: or iii) Advanced Interstitial lung Disease (ILD), defined by carbon monoxide transfer factor (TLCO/DLCO) level of <40% or FVC <50% predicted [18]. Patients were excluded if they had a clinician estimated life-expectancy of less than one month, lacked capacity to consent, or were unable to complete the survey in English.

### Recruitment and data collection

We recruited between July 2020 and January 2021 during the first year of the Covid-19 pandemic. There were twelve participating centres across England including eight acute NHS trusts, three hospices, and a national charity. Recruitment settings included: hospital medical, respiratory or oncology wards; outpatient lung cancer or respiratory clinics; and hospice/palliative care inpatient, outpatient, and community services. The study was also advertised through the British Lung Foundation (BLF) members’ forum.

Clinical staff identified and approached potential participants at a routine face to face or virtual consultation. Names of interested patients were given to the local researcher via secure NHS email, who contacted participants by telephone to explain the study in more detail. If in agreement, participants were sent a patient information sheet and consent form in the post. Informed consent and the baseline questionnaire were completed either face to face or verbally over the telephone.

Prospective data collection consisted of seven timepoints for repeated measures: baseline and then each month for the following six months. A self-reported postal survey was used for follow-up, with the option to complete the questionnaire over the telephone if preferred. As standard, participants had the opportunity to withdraw at any time. Participants who did not return a questionnaire and were uncontactable were considered lost to follow-up and withdrawn from the study. All personal data were managed according to the principles established in the Data Protection Act 2018 [19].

### Patient and public involvement

The public engagement forum at the Cicely Saunders Institute was utilized to engage patients and members of the public in the study design. This included: facilitating the choice of outcome measures; language for patient facing documents and clinical materials; and critical reflection on the data collection process and tools prior to roll out.

### Outcomes

Disability in basic ADLs was measured using the Barthel index which consists of ten items (bowel incontinence, toilet use, grooming, feeding, mobility, bladder incontinence, dressing, bathing, stairs, transfers) [20]. Each item has a range of two to four categorical responses rated on a 0-4 scale, ranging from dependent/unable, to minor help, major help, or independent, depending on the activity. A summary score ranges from 0-20 where a score of twenty represents no disability, and a score of zero indicates fully dependent [20]. Disability in instrumental ADLs was measured using the Lawton Brody IADL scale. This is an eight-item categorical measure (ability to use the telephone, shopping, food preparation, housekeeping, laundry, mode of transportation, responsibility for own medication, ability to manage finances) [21]. Each item has a range of three to five responses ranging from fully dependent (0), through variations of partial dependency (0), to fully independent (1). A summary score ranges from zero (low function, dependent) to eight (high function, independent) [21]. On both measures a lower score indicates greater ADL disability.

### Participant demographics and explanatory variables

Demographic data and participant characteristics were collected at baseline only. These included diagnosis and staging, cancer treatment, oxygen therapy, age, gender, ethnicity, education, living status, current location, and carer support. In response to the Covid-19 pandemic we also collected: time spent in physical and social isolation (in months); change in physical activity since physically or socially isolating, measured using a five-point Likert scale; and confidence to receive social support using the Chronic Disease Self-Efficacy Scale ‘confidence in managing chores, receiving social support and participation in society subscale’ [22,23].

Explanatory variables collected monthly, included symptom severity (Palliative care Outcomes Scale – Symptoms (POS-S)) [24], multi-morbidity (Charlson Co-morbidity Index score) [25], functional performance (Australian Karnofsky Performance Status (AKPS))[26], service utilization (Clinical Service Receipt Inventory (CSRI)), and use of assistive devices (self-reported). Difficulty in managing daily activities was measured using the World Health Organisation Disability Assessment Schedule (WHODAS-2⋅0), consisting of six domains (cognition, mobility, self-care, getting along with others, household activities, societal participation) [27]. Domain items were scored on a scale of activity difficulty, ranging from one to five: none (1), mild (2), moderate (3), severe (4), and extreme or cannot do (5). Domain scores were totalled to produce a WHODAS-2⋅0 summary score, where 32 reflects no difficulty and 160 extremely difficult [27]. The WHODAS-2⋅0 mobility domain [28] consists of five mobility tasks (standing for long periods, standing up from sitting down, moving around inside the home, getting out of your home, walking a long distance), rated on the five-point scale. A summary score ranges from five to twenty-five, where a score of six or more indicates mobility limitation.

### Sample size

For the purpose of this analysis, a sample size of 200 was sufficient to achieve a precision of at least 8% in the estimation of prevalence of ADL disability in the population of interest, based on assumed prevalence to be around 50% [5,29], accounting for an estimated attrition rate of 40% [30]. The sample was also sufficient to adjust for up to twenty co-variables in planned multiple variable regression analysis [31].

### Statistical analysis

To examine change over repeated timepoints, participants who completed three or more timepoints were included in the longitudinal analysis. As the data was not normally distributed, descriptive statistics using the Mann-Whitney-u test for continuous variables and chi-square test of independence for categorical variables were selected [32]. These were used to describe participant characteristics and to compare demographic characteristics of participants included in the longitudinal sample and those who were not. Summary statistics were used to determine trajectories of ADL disability at group level, by reporting median [IQR] at each timepoint and plotted over time, separately for basic and instrumental ADLs

Individual trajectories of ADL disability were explored using visual graphical analysis (VGA) [33]. To categorise individual trajectories, each participants trajectory was prepared for inspection on a line graph using identical scales separately for basic and instrumental ADLs, and eyeballed by two independent researchers, to enhance reliability [33]. As missing data was most likely to be “not missing at random” due to death or ill health, imputation methods were not appropriate. Participants completing three or more time points were included in the analysis, as these data would provide important information for the description of trajectories. On individual trajectory plots, missing timepoints were not connected to illustrate where data were missing.

Change at each time point in an individual’s trajectory is indicated by a change of ≥2 on the Barthel Index [20] and ≥0⋅5 on the Lawton Brody Instrumental ADL scale [34] to identify change in basic ADL and instrumental ADL disability trajectories, respectively. Changing trajectories could either change in one direction (only increasing in score or decreasing in score) or fluctuate. The individual line graphs were independently grouped into four ADL disability trajectory groups (increasing, decreasing, fluctuating, and stable (includes no disability and unchanging disability)), defined by distinct rules as outlined in Table 1.

**Table 1.** Categorisation of individual trajectory groups of basic and instrumental ADL disability.

Trajectory Sub-group	Variable criteria	
	Basic ADLs (Barthel Index: 0–20)	Instrumental ADLs (Lawton Brody IADL Scale: 0–8)
Increasing disability	Decrease in score over 6-months (including downwards fluctuation) of ≥2.	Decrease in score over 6-months (including downwards fluctuation) of ≥0⋅5.
Decreasing disability	Increase in score over 6-months (including upwards fluctuation) of ≥2.	Increase in score over 6-months (including upwards fluctuation) of ≥0⋅5.
Fluctuating disability	At least one increase and one decrease (or vice versa) between any two timepoints of ≥2.	At least one increase and one decrease (or vice versa) between any two timepoints of ≥0⋅5.
Stable disability	Difference in score between first and last recorded timepoint is <2 (includes no disability).	Difference in score between first and last recorded timepoint is <0⋅5 (includes no disability).

A lower score on the Barthel Index or Lawton Brody IADL Scale indicates greater disability. A meaningful clinical difference is based on a change of ≥2 on the Barthel Index and ≥0.5 on the Lawton Brody IADL Scale.

Univariable logistic regression was used to identify baseline associations with the increasing disability trajectory, separately for basic and instrumental ADLs. Participants included in this analysis had either an increasing or stable disability trajectory, where the constant variable was increasing disability compared to stable disability. Significance levels were set at 0⋅01 to account for multiple testing [35]. Covariables considered for the multiple-variable logistic regression model were selected based on findings from the systematic review [13], or if they had a p-value of ≤0⋅01 in the univariable analysis. Considered covariables included the Charlson Comorbidity Index Score, functional performance status (AKPS), Barthel Index and Lawton Brody Instrumental ADL total scores, difficulty managing daily activities in WHODAS-2⋅0 or sub-domains, cancer treatment, oxygen therapy, age, gender, living alone, ethnicity, education level, reduced physical activity, months spent in physical and social isolation, and service use. They were eliminated from the models if there was collinearity between explanatory and/or confounding variables identified in scatter plots between continuous variables. The strength of the association was described using adjusted Odd Ratios and 95% confidence interval (95% CIs.). All statistical analyses were completed with Stata 16.

#### Role of funding source

The funder of the study had no role in the study design, data collection, data analysis, data interpretation, or writing of the report. The corresponding author had full access to all the data in the study and had the final responsibility for the decision to submit the report for publication.

## Results

### Participant flow

**Figure 1.**
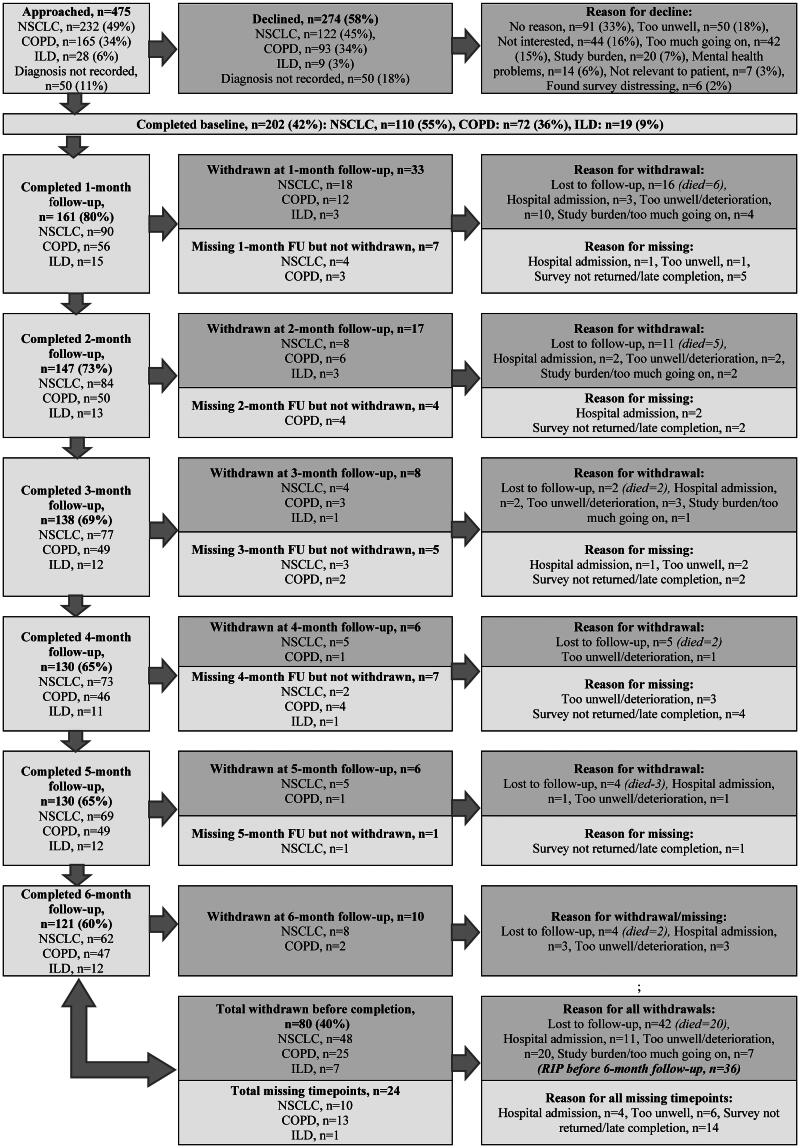
Study flow of recruitment, follow-up, and attrition. NSCLC: non-small cell lung cancer; COPD: chronic obstructive pulmonary disease; ILD: interstitial lung disease; FU: follow-up.

There were 201 participants recruited between March 2020 and January 2021, with a diagnosis of NSCLC (*n* = 110), COPD (*n* = 72), ILD (*n* = 19). The study flow of follow-up, attrition and missing timepoints with reasons is presented in Figure 1. The overall attrition to six months was 80 participants (40%), most of whom had a primary diagnosis of NSCLC (*n* = 48, 60%). Attrition was greatest at 1-month follow-up (*n* = 33, 16%). Medical notes were checked at six-month follow-up which clarified that 36 (45%) participants who withdrew or were lost to follow-up had died within six months following consent (before six-month follow-up), which reflects the general mortality of this population. Most participants who died had NSCLC (*n* = 25, 69%).

### Characteristics of study sample

One-hundred-and fifty-one participants contributed data at ≥3 timepoints during six-month follow-up and were included in the longitudinal analysis (Table 2). 85 (56%) participants had NSCLC, and 66 (44%) had COPD or ILD. At baseline, the cross-sectional analysis [7] identified over half of participants had disability in ADLs, where instrumental ADLs (70%) were more greatly affected than basic ADLs (52%). Nearly all participants were physically and socially isolating as a direct consequence of the Covid-19 pandemic, and 63% of participants reported reduced physical activity outdoors. Most participants were able to care for themselves, which was reflected by the AKPS score (median: 70 [IQR:60-80]). However, their confidence to receive social support was extremely low, at an average of two out of ten on the Chronic Disease Self-Efficacy Scale. The Palliative Care Outcome Scale scores indicated mild effects of symptoms at baseline (median: 9 [IQR:5–14]). The longitudinal sample had a significantly higher AKPS, lower symptom severity, less disability in instrumental ADLs, and lower use of ADL assistive devices than those not included in this analysis. These participants also presented with less difficulty in daily activities across all WHO-DAS 2⋅0 domains, had lower AKPS, and greater symptom severity.

**Table 2.** Differences in baseline characteristics between participants included in the longitudinal analysis (completed ≥3 timepoints) and participants excluded from the longitudinal analysis (completed <3 timepoints).

Participant characteristics and outcomes at baseline	Whole sample *n* = 201	Completed 3 or more timepoints (*n* = 151)	Completed <3 timepoints (*n* = 50)	Difference between groups (*p* value)
**Health-related factors**				
NSCLC, *n* (%)	110 (55%)	85 (56%)	25 (50%)	0⋅44
COPD or ILD, *n* (%)	91 (45%)	66 (44%)	25 (50%)	
Stage III disease, *n* (%)	80 (40%)	63 (42%)	7 (34%)	0⋅27
Stage IV disease, *n* (%)	121 (60%)	88 (58%)	33 (66%)	
Charlson comorbidity Index score, median [IQR]	7 [3–10]	7 [3–11]	7 [3–9]	0⋅83
**Body Functions and Structures**				
Australian Karnofsky Performance Status (AKPS), median [IQR]	70 [60–80]	70 [60–80]	60 [50–70]	**0**⋅**0002**
Symptom severity (Palliative care Outcome Scale-symptoms), median [IQR}	10 [5.5–15]	9 [5–14]	13 [8–19]	**0**⋅**0002**
Receiving cancer treatment, n (%)	100 (50%)	78 (52%)	22 (45%)	0⋅51
On oxygen therapy, n (%)	40 (20%)	29 (19%)	11 (23%)	0⋅76
**Activity and participation**				
Total Barthel Index score (BADLs), median [IQR]	19 [17–20]	20 [15–20]	19 [16–20]	0⋅14
Lawton Brody Instrumental ADL score (IADLs), median [IQR]	7 [5–8]	7 [5-8]	5.5 [3–7]	**0**⋅**002**
WHODAS 2⋅0 Summary score, median [IQR]	57 [46–79]	53.5 [44–72]	82 [53–92]	**<0**⋅**0001**
*Cognition,* median [IQR]	7 [6–10]	7 [6–9]	9 [7–16]	**<0**⋅**0001**
*Mobility,* median [IQR]	13 [7–17]	12 [7–16]	16 [11–19]	**0**⋅**0001**
*Self-Care,* median [IQR]	5 [4–9]	4 [4–8]	8 [4–10]	**0**⋅**0001**
*Getting along with people*, median [IQR]	9 [4–13]	8 [5–10]	9 [7–14]	**0**⋅**002**
*Household activities,* median [IQR]	9 [4–13]	8 [4–12]	14 [8–20]	**<0**⋅**0001**
*Societal participation,* median [IQR]	17 [12–21]	16 [11–20]	20 [14–24]	**0**⋅**001**
**Personal factors**				
Age, median [IQR]	69 [63–75]	70 [64–76]	68 [61–74]	0⋅12
Female, *n* (%)	91 (45%)	65 (43%)	26 (52%)	0⋅27
White British, *n* (%)	191 (95%)	143 (95%)	48 (96%)	0⋅72
Education above secondary school, *n* (%)	93 (46%)	74 (49%)	18 (36%)	0⋅11
CDSE: Confidence to receive help, median [IQR]	2 [1.5–3]	2 [1.5–3]	2 [1–3]	0⋅17
**Environmental factors**				
Lives alone, n (%)	68 (34%)	45 (30%)	23 (46%)	0⋅04
Inpatient/residential care, *n* (%)	4 (2%)	1 (1%)	3 (6%)	0⋅02
Property with stairs, *n* (%)	144 (72%)	110 (73%)	34 (68%)	0⋅51
Formal caregiver, *n* (%)	29 (14%)	18 (12%)	11 (22%)	0⋅08
Informal caregiver, *n* (%)	112 (56%)	82 (55%)	30 (60%)	0⋅62
Physiotherapy input within the last month, *n* (%)	20 (10%)	14 (9%)	6 (12%)	0⋅76
Occupational therapy input within the last month, *n* (%)	10 (5%)	5 (3%)	5 (10%)	0⋅15
Receiving community (or hospice) palliative care, *n* (%)	48 (24%)	31 (21%)	17 (34%)	0⋅05
Total use of ADL devices, median [IQR]	1 [0–4]	1 [0–3]	3 [1–5]	**0**⋅**001**
Have spent time in physical and social isolation, *n* (%)	194 (97%)	146 (97%)	49 (98%)	0⋅64
Reduced physical activity outside the home, *n* (%)	129 (65%)	95 (63%)	34 (69%)	0⋅6

NSCLC: non-small-cell lung cancer; COPD: chronic obstructive pulmonary disease; ILD: Interstitial lung disease; ADL: activities of daily living; BADL: Basic activities of daily living; IADL: instrumental activities of daily living; WHODAS2⋅0: World Health Organization disability assessment Schedule; CDSE: Chronic Disease Self-Efficacy subscale; Reduced physical activity includes responses of “little less” or a “lot less”; IQR: inner quartile range; Statistical comparisons between the two groups was conducted using the Mann Whitney-*U* test for continuous variable and the Chi square test for categorical variables.; the significance level is set at *p* ≤ 0⋅01.

### ADL disability trajectories

For participants overall, the median disability in basic ADLs is shown to fluctuate by 0⋅5 to 1 point on the Barthel index between baseline (median 20 [1QR 17–20]), one-month follow-up (median 19 [1QR 17–20]), four-month follow-up (median 20 [1QR 18–20]), and six-month follow-up (median 19.5 [1QR 17–20]). Disability (median) in instrumental ADLs did not change over six months and was maintained at a median [IQR] of 7 [5–8] at all follow-up timepoints.

**Figure 2.**
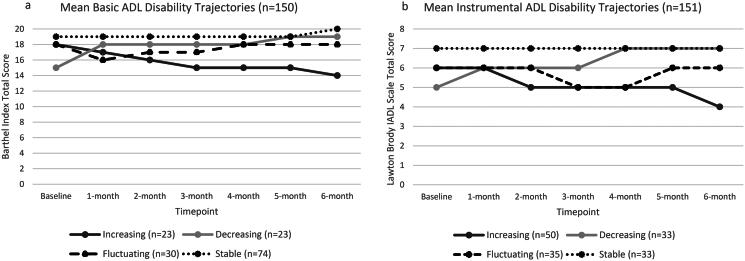
Mean ADL disability trajectories. ADL: activities of daily living; Lower score on Barthel Index or Lawton Brody Instrument ADL (IADL) Scale: greater disability.

Figure 2 illustrates the mean ADL disability trajectory in each group for both BADL and IADL, with reference to the total number of participants presenting with that trajectory. Half of participants had basic ADL disability trajectories categorised as “stable” (*n* = 74, 50%), and the remainder had increasing (*n* = 23, 15%), decreasing (*n* = 23, 15%), and fluctuating (*n* = 30, 20%) disability trajectories. For instrumental ADL disability, increasing disability (*n* = 50, 33%) was the most common trajectory, with little difference between the decreasing (*n* = 33, 22%), fluctuating (*n* = 35, 23%) and stable (*n* = 33, 22%) trajectory groups.

### Predictors of increasing ADL disability trajectories

The univariable logistic regression analysis identified significant factors associated with the increasing disability trajectory for basic and instrument ADLs and can be found in Supplementary Material C (Tables S1 and S2). The multi-variable logistic regression model (*n* = 82) (Table 3), adjusting for these factors showed that difficulty mobilising (odds ratio, 1.41 [95%CI: 1⋅14–1.74], *p* = 0.002) was associated with an increasing instrumental ADL disability trajectory. No independent predictors of increasing disability trajectories in basic ADLs were identified.

**Table 3.** Multiple variable regression analysis of factors predicting an increasing disability trajectory in basic and instrumental ADLs, compared to a stable disability trajectory.

Participant characteristics and outcomes at baseline	Basic ADLs (*n* = 97)			Instrumental ADLs (*n* = 82)		
	odds ratio	95% CI	*p*-value	odds ratio	95% CI	*p*-value
**Health-related factors**						
*NSCLC*	0⋅82	0⋅72 − 9.38	0⋅87	6⋅02	1⋅01 – 35⋅84	0⋅05
Charlson comorbidity Index score	0⋅9	0⋅72 − 1⋅11	0⋅33	⋅⋅	⋅⋅	⋅⋅
**Body Functions and Structures**						
Symptom severity (Palliative care Outcome Scale-symptoms)	1⋅05	0⋅93 − 1⋅18	0⋅72	1⋅03	0⋅89 − 1⋅2	0⋅69
Receiving cancer treatment	6⋅57	0⋅54–79⋅83	0⋅14	⋅⋅	⋅⋅	⋅⋅
**Activity and participation**						
Total Barthel Index score (BADLs)	1⋅06	0⋅72–1⋅⋅55	0⋅77	0⋅86	0⋅62 − 1⋅21	0⋅4
Lawton Brody Instrumental ADL score (IADLs)	0⋅92	0⋅59–1⋅47	0⋅75	1⋅38	0⋅8 − 2⋅37	0⋅25
Mobility limitation (WHODAS-2⋅0-mobility)	1⋅13	0⋅96 − 1⋅33	0⋅15	1⋅41	1⋅14 − 1⋅74	0⋅002
**Personal Factors**						
Age	1⋅07	0⋅99 − 1⋅15	0⋅09	0⋅99	0⋅93 − 1⋅05	0⋅76
Female	0⋅89	0⋅26 − 3⋅08	0⋅86	0⋅98	0⋅29 − 3⋅25	0⋅96
**Environmental factors**						
Lives alone	1⋅1	0⋅27– 4⋅49	0⋅9	0⋅49	0⋅14 − 1⋅72	0⋅27
Receiving community (or hospice) palliative care	3⋅05	0⋅54 − 17⋅21	0⋅21	–	–	–
Total number of ADL devices	1⋅34	0⋅89 − 3⋅02	0⋅17	1⋅06	0⋅68 − 1⋅64	0⋅81
Reduced physical activity outdoors	–	–	–	2⋅02	0⋅62 − 6⋅61	0⋅25
**Constant (Increasing disability trajectory)**	**0**⋅**00009**	**2**⋅**75e-09 − 3**⋅**02**	**0**⋅**08**	**0**⋅**02**	**3**⋅**47e-06 − 148**⋅**07**	**0**⋅**4**

ADL: activities of daily living; NSCLC: non-small cell lung cancer; BADL: basic activities of daily living; IADL: instrumental activities of daily living; WHODAS: world health organization disability assessment schedule; ⋅⋅ not included in the model.

## Discussion

### Main findings

This longitudinal study of 201 participants with advanced respiratory disease found: i) Individual trajectories show wide variation in ADL disability, missed at group-level, which can be categorised into one of four identified trajectories of ADL disability: increasing, decreasing, fluctuating, or stable; ii) ADL disability arises from a combination of personal, health-related and environmental factors; iii) Mobility limitation was an independent predictor of increasing disability in instrumental ADLs over the subsequent six months.

### Contributions to the literature

#### i) variation in individual trajectories of ADL disability

Individual ADL disability trajectories in this study show that people with a diagnosis of NSCLC or COPD or ILD can follow any one of the four main types of ADL disability trajectory (increasing, decreasing, fluctuating, or stable). This highlights the importance of incorporating individual trajectories over time and using a longitudinal approach [33]. This individual variation raises some speculation over findings from a systematic review of trajectories of terminal decline [36], where organ failure including respiratory disease follow a fluctuating trajectory and cancer follows a stable trajectory followed by sudden decline, as first suggested in retrospective study by Lunney et al. 2003 [37]. Our prospective findings may reflect the more chronic nature of NSCLC resulting from advances in cancer treatment keeping patients on a stable, fluctuating, or even improving disability trajectory for a longer period, prior to sudden decline. Within non-malignant respiratory disease there can be difference in natural course of disease, oxygen saturation, and co-morbidity profiles.

Using disease-based criteria for study inclusion attempted to identify participants at a stage when their disease was advanced as an indication of decline, but this is likely to increase the heterogeneity of the study population. In terms of prognosis some participants may be close to death, and some may not. This potentially leads to a range and diversity of symptoms and functional limitation. Changes over time may be effectively diluted by this heterogeneity and eventual deterioration be less easy to identify using a prospective approach. Population-level ageing studies have shed some light on understanding disability over a longer period, using latent trajectory modelling. The Precipitating Events Project (PEP Project) of 747 community dwelling elders [38] assessed disability in ADLs monthly and drew trajectories in the last year of life [39]. Lunney’s recent work on mobility trajectories in a population-based sample of 3075 participants aged over seventy [40], carried out 6-monthly assessments over three years. Despite differences in study design and data collection, both these studies have challenged the usefulness of clinical groupings for categorising disability trajectories. The authors argue that clinical diagnosis does not adequately predict the trajectory of ADL disability or decline in mobility in the last years of life.

#### ii) complexity of ADL disability

The cohort study examined factors that predict a subsequent increasing disability trajectory in basic or instrumental ADLs, and found health-related, personal, and environmental factors are determinants, fitting the WHO-ICF framework [4]. This confirms that disability is complex and generally the product of a combination of factors. This is supported by baseline data which identified significant associations between increased symptom severity and greater time spent in physical and social isolation with ADL disability [7].

Increased symptom severity was associated with disability in basic and instrumental ADLs. A prospective analytical study of 638 patients referred to a home care support team, supports this finding, where an individual’s ability to manage at home prompting a referral was linked to symptom burden [41]. During the last year of life, symptoms restricting disability are common, and patients with a greater burden of restricting symptoms and number of disabilities in ADLs are increasingly likely to receive hospice care [42]. Higher symptom severity is also associated with a housebound status, reducing a persons’ ability to carry out activities involving socialising and participating in the community [43]. This highlights the importance of understanding how symptoms may be restricting an individual’s function when assessing disability.

Nearly all participants were physically and socially isolating during the Covid-19 pandemic and at the same time they did not feel they received the social support they required. This meant that participants may have reported being independent in a particular activity but still had difficulty completing that activity, which would be undetected by a measure of dependency [7]. In addition, reduced physical activity was aggravated by physical and social isolation, known to be associated with functional decline [44]. This highlights the impact Covid-19 restrictions have had on this population, initially portrayed as feelings of vulnerability about catching Covid-19 [45], even after restrictions were lifted or removed. This reduced confidence to participate in normal daily activities transpires as deconditioning and functional impairment, leading to disability [46,47].

#### Iii) Predictors of increasing ADL disability

The study highlights mobility limitation as an independent predictor of increasing disability. Mobility limitation may be viewed as a “tipping point” or “transition” for declining function, identifying a critical time where rehabilitation interventions can be targeted to provide most benefit on ADL independence. This is supported by a systematic review in older adults [48], which found that identifying risk for mobility limitation can be accomplished through routine screening, and functional deficits and environmental barriers can be addressed with proprioceptive exercise and assessing a patients ability to adapt to their physical environment using provision and practice of mobility devices, leading to improved function, safety, and quality of life.

The concept of early identification of functional decline aligns with Gore et al. [49], who proposed the framework of, compression of functional decline (CFD), based on the latest understanding of the hierarchy of age-related functional decline [50,51]. In the CFD model, the trajectory of disability in ADLs represents the stages through which an individual progresses through the disablement process, encountering difficulty with, or inability to perform each of the fifteen activities in the hierarchy, independently [49]. The model highlights that use of preventative interventions at the right time, can potentially compress the trajectory of disability into a shorter timeframe.

### Clinical implications

In advanced respiratory disease, it is important to identify risk of disability to prompt referral for intervention. Clinical conditions are only one of many factors that influence the course of functional decline and disability, suggesting a need for interventions targeting function irrespective of diagnosis or prognosis [4,40]. Current preventative rehabilitation models are disease specific (e.g., Pulmonary rehabilitation or pre-habilitation in cancer) and may not always be appropriate or accessible for patients approaching the end of their life. Rehabilitation services specialising in advancing disease are few and far between, and are often specific to inpatient or outpatient hospice services, which reach the minority of this population [52]. Generalist rehabilitation services within the community (visiting patients at home) often contain barriers such as long waiting lists delaying timely intervention, lack of understanding of the needs of a deteriorating patient, or reduced confidence within the team to manage their needs. This means, people with advancing disease often don’t receive rehabilitation or receive services that are reactive to functional decline rather than preventative, e.g., intermediate care post hospital discharge or urgent community response teams [53].

Enabling the advanced disease population to access a preventative model of rehabilitation, considering forthcoming decline in function, could help maintain independence or prevent or delay potential disability in ADLs. This would move rehabilitation in advanced disease away from reactive crisis management. Preventing a potential crisis such as a fall or hospital admission may not only help improve a patient’s quality of life but reduce the burden on informal carers and health and social care services. It could be argued that a preventative model of rehabilitation would benefit patients presenting with early signs of functional deficit, particularly older people [48], and not limited to those with advanced disease.

This study has identified that mobility limitation in advanced respiratory disease predicts increasing disability, and this could therefore serve to identify a population in which to target preventative interventions, regardless of specific diagnosis or prognosis. Mobility limitation could be used to identify the right time to refer to rehabilitation services, using available measures [54]. However, careful work is required to understand thresholds for screening (e.g., severity of symptoms, and time spent in physical and social isolation), as well as to select appropriate screening tools and develop cut-offs for referral criteria (e.g., mobility limitation tools, items, or questions). Decisions regarding choice of tools and/or thresholds, may also depend on what available clinical services can offer, and adaptability may be essential for such a system to work effectively in clinical care.

### Study strengths and limitations

A prospective cohort study design was the most appropriate design choice to distinguish change in ADL disability over time and predicting factors [55]. Trajectories were drawn from patient-reported outcomes, at regular monthly intervals, allowing for identification of short-term changes. We recruited a large representative sample of patients with advanced respiratory disease across multiple sites to increase generalisability of the findings. Potential bias includes varying time of individual data collection, enforced physical and social isolation during the Covid-19 pandemic with fluctuating guidance over the recruitment time-period, and response or recall from self-reported measures. ADL disability trajectories were descriptive rather than quantifiable, and healthy patient bias in the data may underestimate the effect some factors have on disability. Participants who were not included in the longitudinal analysis due to withdrawal or loss to follow-up presented with greater symptom severity and difficulty with daily tasks at baseline, which may plausibly strengthen the relationship if these participants were included in the analysis.

Finally, identified associations of increasing disability were limited by variables collected in the study. Respiratory function tests were not performed, as all eligible participants had an advanced disease diagnosis which is based on respiratory function, therefore we did not feel it was necessary to repeat these tests in order to limit study burden for participants and increase study compliance and completion. Symptom burden using the POS-S was analysed as a summary score, but it may have been more relevant to use the dyspnoea item of the POS-S due to the known effect of breathlessness on functional capability [56]. It may have been pertinent to collect information regarding exacerbation, which is highly relevant in a respiratory disease population and potentially related to disability. A measure of sarcopenia may have been useful to identify cancer cachexia which may also have an impact on function and participation in daily activities.

## Conclusion

Disability in ADLs affects over half of people with advanced respiratory disease with wide individual variability in basic and extended ADL disability over time. An increasing disability trajectory can be predicted by mobility limitation, which could be used to prompt referral to rehabilitation services in this population.

## Supplementary Material

Supplemental Material

## Data Availability

Individual participant data will not be available.
