# Supplementary material for: Predictors of increasing disability in activities of daily living among people with advanced respiratory disease: a multi-site prospective cohort study, England UK
Source: Disabil Rehabil. 2023 Dec 10;46(20):4735–44. doi: 10.1080/09638288.2023.2288673 (PMC11441397; doi:10.1080/09638288.2023.2288673)
Supplement: Supplemental Material [file IDRE_A_2288673_SM7471.zip › Fettes Predictors paper Supplementary material A STROBE checklist.docx]

STROBE Statement—checklist of items that should be included in reports of observational studies

|  | Item No. | Recommendation | Page  No. | Relevant text from manuscript |
| --- | --- | --- | --- | --- |
| **Title and abstract** | 1 | (*a*) Indicate the study’s design with a commonly used term in the title or the abstract | Submitted copy – page 2 | Multi-site prospective cohort study |
|  |  | (*b*) Provide in the abstract an informative and balanced summary of what was done and what was found | Submitted copy – page 2 | See whole abstract |
| Introduction | | | |  |
| Background/rationale | 2 | Explain the scientific background and rationale for the investigation being reported | Submitted copy – page 4 | Paragraphs 4 and 5 of introduction |
| Objectives | 3 | State specific objectives, including any prespecified hypotheses | Submitted copy – page 4 | This study aims to describe the course of disability in ADLs over time and identify predictors of increasing disability in ADLs to inform clinical care for people with advanced respiratory disease. |
| Methods | | | |  |
| Study design | 4 | Present key elements of study design early in the paper | Submitted copy – page 4 | ‘Study design’ of Materials and methods |
| Setting | 5 | Describe the setting, locations, and relevant dates, including periods of recruitment, exposure, follow-up, and data collection | Submitted copy – page 5 | ‘Recruitment and data collection’ section of Materials and methods |
| Participants | 6 | (*a*) *Cohort study*—Give the eligibility criteria, and the sources and methods of selection of participants. Describe methods of follow-up  *Case-control study*—Give the eligibility criteria, and the sources and methods of case ascertainment and control selection. Give the rationale for the choice of cases and controls  *Cross-sectional study*—Give the eligibility criteria, and the sources and methods of selection of participants | Submitted copy – page 4-5 | ‘Participants’ section of Materials and methods and last two paragraphs of ‘Recruitment and data collection’ section |
|  |  | (*b*) *Cohort study*—For matched studies, give matching criteria and number of exposed and unexposed  *Case-control study*—For matched studies, give matching criteria and the number of controls per case | NA |  |
| Variables | 7 | Clearly define all outcomes, exposures, predictors, potential confounders, and effect modifiers. Give diagnostic criteria, if applicable | Submitted copy – page 6-7 | See ‘Outcomes’ and ‘Participant demographics and explanatory variable’sections of of Materials and methods |
| Data sources/ measurement | 8* | For each variable of interest, give sources of data and details of methods of assessment (measurement). Describe comparability of assessment methods if there is more than one group | Submitted copy – page 6-7 | See ‘Outcomes’ and ‘Participant demographics and explanatory variable’ sections of of Materials and methods |
| Bias | 9 | Describe any efforts to address potential sources of bias | Submitted copy – page 6 | *See ‘Patient and Public Involvement* section’ of Materials and methods |
| Study size | 10 | Explain how the study size was arrived at | Submitted copy – page 7 | See ‘Sample Size’ section of Materials and methods |

Continued on next page

| Quantitative variables | 11 | Explain how quantitative variables were handled in the analyses. If applicable, describe which groupings were chosen and why | Submitted copy – page 8-9 | See ‘Statistical Analysis’ section of section of Materials and methods |
| --- | --- | --- | --- | --- |
| Statistical methods | 12 | (*a*) Describe all statistical methods, including those used to control for confounding | Submitted copy – page 8-9 | See ‘Statistical Analysis’ section of section of Materials and methods |
|  |  | (*b*) Describe any methods used to examine subgroups and interactions | Submitted copy – page 8-9 | See ‘Statistical Analysis’ section of section of Materials and methods |
|  |  | (*c*) Explain how missing data were addressed | Submitted copy – page 8 | See paragraph 2 of ‘Statistical Analysis’ section of section of Materials and methods |
|  |  | (*d*) *Cohort study*—If applicable, explain how loss to follow-up was addressed  *Case-control study*—If applicable, explain how matching of cases and controls was addressed  *Cross-sectional study*—If applicable, describe analytical methods taking account of sampling strategy | Submitted copy – page 8 | See paragraph 2 of ‘Statistical Analysis’ section of section of Materials and methods |
|  |  | (*e*) Describe any sensitivity analyses | NA |  |
| Results | | | | |
| Participants | 13* | (a) Report numbers of individuals at each stage of study—eg numbers potentially eligible, examined for eligibility, confirmed eligible, included in the study, completing follow-up, and analysed | Submitted copy – page 10 | See ‘Participant Flow’ section of section of Results |
|  |  | (b) Give reasons for non-participation at each stage | Submitted copy – page 10 | See ‘Participant Flow’ section of section of Results, and Figure 1: Study flow of recruitment, follow-up, and attrition |
|  |  | (c) Consider use of a flow diagram | Submitted copy – page 10 | See Figure 1: Study flow of recruitment, follow-up, and attrition |
| Descriptive data | 14* | (a) Give characteristics of study participants (eg demographic, clinical, social) and information on exposures and potential confounders | Submitted copy – page 10 | See ‘Characteristics of study sample’ section of Results |
|  |  | (b) Indicate number of participants with missing data for each variable of interest | Submitted copy – page 11 | See Table 2: Differences in characteristics between participants included in the longitudinal analysis (completed ≥3 timepoints) and participants excluded from the longitudinal analysis (completed <3 timepoints)], in Results |
|  |  | (c) *Cohort study*—Summarise follow-up time (eg, average and total amount) | Submitted copy – page 11 | See Table 2: Differences in characteristics between participants included in the longitudinal analysis (completed ≥3 timepoints) and participants excluded from the longitudinal analysis (completed <3 timepoints)], in Results |
| Outcome data | 15* | *Cohort study*—Report numbers of outcome events or summary measures over time | Submitted copy – page 11 | See ‘ADL disability trajectories’  section of Results |
|  |  | *Case-control study—*Report numbers in each exposure category, or summary measures of exposure | *NA* | NA |
|  |  | *Cross-sectional study—*Report numbers of outcome events or summary measures | *NA* | NA |
| Main results | 16 | (*a*) Give unadjusted estimates and, if applicable, confounder-adjusted estimates and their precision (eg, 95% confidence interval). Make clear which confounders were adjusted for and why they were included | Submitted copy – page 12 | See ‘Predictors of increasing ADL disability trajectories’ section of Results |
|  |  | (*b*) Report category boundaries when continuous variables were categorized | Submitted copy – page 12 | See ‘Predictors of increasing ADL disability trajectories’ section of Results |
|  |  | (*c*) If relevant, consider translating estimates of relative risk into absolute risk for a meaningful time period | NA |  |

Continued on next page

| Other analyses | 17 | Report other analyses done—eg analyses of subgroups and interactions, and sensitivity analyses | NA |  |
| --- | --- | --- | --- | --- |
| Discussion | | | | |
| Key results | 18 | Summarise key results with reference to study objectives | Submitted copy – page 12 | See ‘Main finding’ section of Discussion |
| Limitations | 19 | Discuss limitations of the study, taking into account sources of potential bias or imprecision. Discuss both direction and magnitude of any potential bias | Submitted copy – page 15-16 | *See ‘Study strengths and limitations’* section of Discussion |
| Interpretation | 20 | Give a cautious overall interpretation of results considering objectives, limitations, multiplicity of analyses, results from similar studies, and other relevant evidence | Submitted copy – page | *See ‘Contributions to the literature’*  section of Discussion |
| Generalisability | 21 | Discuss the generalisability (external validity) of the study results | Submitted copy – page 15-16 | *See ‘Study strengths and limitations’* section of Discussion |
| Other information | |  | | |
| Funding | 22 | Give the source of funding and the role of the funders for the present study and, if applicable, for the original study on which the present article is based | Submitted copy – page 17 | See Acknowledgements section |

*Give information separately for cases and controls in case-control studies and, if applicable, for exposed and unexposed groups in cohort and cross-sectional studies.

**Note:** An Explanation and Elaboration article discusses each checklist item and gives methodological background and published examples of transparent reporting. The STROBE checklist is best used in conjunction with this article (freely available on the Web sites of PLoS Medicine at http://www.plosmedicine.org/, Annals of Internal Medicine at http://www.annals.org/, and Epidemiology at http://www.epidem.com/). Information on the STROBE Initiative is available at www.strobe-statement.org.
