# Supplementary material for: Predictors of increasing disability in activities of daily living among people with advanced respiratory disease: a multi-site prospective cohort study, England UK
Source: Disabil Rehabil. 2023 Dec 10;46(20):4735–44. doi: 10.1080/09638288.2023.2288673 (PMC11441397; doi:10.1080/09638288.2023.2288673)
Supplement: Supplemental Material [file IDRE_A_2288673_SM7471.zip › Fettes Predictors paper Supplementary material B .docx]

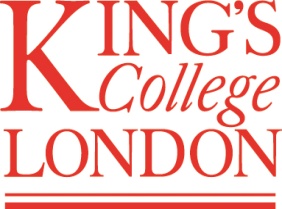


| 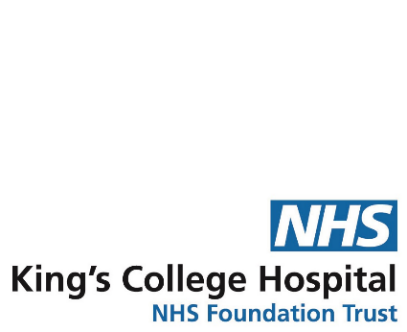**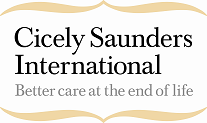**  Comparing disability in activities of daily living over time  among adults with advanced lung cancer or respiratory disease  **during the COVID-19 Pandemic**:  Study Protocol (DIScOVER) |
| --- |

| **Investigators/ Researchers** | **Chief Investigator**:  Dr Matthew Maddocks  Cicely Saunders Institute, Kings College London e-mail: [matthew.maddocks@kcl.ac.uk](mailto:matthew.maddocks@kcl.ac.uk)  Tel: 02078485242  **Co-investigator:**  Lucy Fettes  Cicely Saunders Institute, Kings College London e-mail: [lucy.fettes@kcl.ac.uk](mailto:lucy.fettes@kcl.ac.uk) Tel: 02078485385  Dr Stephen Ashford  Cicely Saunders Institute, Kings College London e-mail: [stephen.ashford@kcl.ac.uk](mailto:stephen.ashford@kcl.ac.uk)  Tel: 02078485564  Prof Irene Higginson  Cicely Saunders Institute, Kings College London e-mail: [irene.higginson@kcl.ac.uk](mailto:irene.higginson@kcl.ac.uk)  Tel: 02078485585 |
| --- | --- |
| **Sponsor**  **Co-Sponsor:** | Prof Reza Razavi, Director of Research Management &  Director of Administration (Health Schools)  Address: Room 5.31, James Clerk Maxwell Building, 57 Waterloo Road, London SE1 8WA  Tel: +44 (0)207 8483224  Email: [reza.razavi@kcl.ac.uk](mailto:reza.razavi@kcl.ac.uk)  Rahman Ahmed, Research & Innovation Governance Manager  Address: King’s College Hospital NHS Foundation Trust, 161 Denmark Hill, LONDON, SE5 8EF.  Tel: 020 3299 6625  e-mail: [rahman.ahmed1@nhs.net](mailto:rahman.ahmed1@nhs.net) |
| **Funder (s):** | National Institute for Health Research |
| **IRAS Reference:** | 271894 |

**Contents**

**Study summary………………………………………………………………………………………………………………………….… 4**

**One Page Summary…………………………………………………………………………………….……………………………….. 5**

**Background and Rationale…………………………………………………………………………………………………………... 6**

**Aims and objectives…………………………………………………………………………………………………………………….. 7**

**Methods………………………………………………………………………………………………………………………………………. 8**

**a) Overview of study design………………………………………………………….……………………………………………… 8**

**b) Study population and sample………………………………………………………………………………………………….. 9**

**c) Inclusion and exclusion criteria…………………………………………………………………………………………………. 9**

**d) Recruitment of participants……………………………………………………………………………………………………… 9**

i) Setting and identification of participants…………………….…………………………………………………… 9

ii) Usual Consent……………………………………………………………………………………………………………….. 10

iii) Remote Consent……………………………………………………………………………………………………………. 11

**e) Data collection schedule…………………………………………………………………………………………………………. 13**

**f) Data collection measures………………………………………………………………………………………………………… 13**

**g) Study Procedures……………………………………………………………………………………………………………………. 15**

i) Pilot phase………………………………………………………………….…………………………………………………… 15

ii) Documentation of participants……………………………………………………………………………………….. 15

**h) Safety and reporting……………………………………………………………………………………………………………….. 16**

i) Steps to prevent harm……………………………………………………………………………………………………… 16

ii) distress protocol…………………………………..………………………………………………………………………… 16

**i) Data handling and management……………………………………………………………………………………………… 17**

**j) Statistical Analysis…………………….……………………………………………………………………………………………. 18**

1. Proposed sample size………………………………………………………………………………………………………… 18
2. Principles of analysis and data usage…………………………………………………………………………………. 19
3. Specific analysis plan…………………………………………………………………………………………………………. 19

**k) Patient and public involvement (PPI)……………………………………………………………….…………………….. 24**

**l) Ethical and regulatory approval………………………………………………………………………………………………. 24**

**m) Dissemination……………………………………………………………………………………………………………………….. 24**

**n) Funding and costings……………………………………………………………………………………………………………….25**

**o) Project timeline……………………………………………………………………………………………………………………… 25**

**References…………………………………………………………………………………………………………………………………. 26**

**STUDY SUMMARY**

| **STUDY OVERVIEW** | |
| --- | --- |
| Full title | Comparing disability in activities of daily living over time, among adults with advanced lung cancer or respiratory disease **during the COVID-19 pandemic** |
| Objectives | **Aim:** To compare and contrast trajectories of disability in activities of daily living (ADLs) over time, among adults with advanced lung cancer or respiratory disease  **Objectives:**  To describe and compare in people with advanced lung cancer or respiratory disease the following:   1. Trajectories of symptom severity and ADL disability over 6 months. 2. Extent to which different disability items of BADL, IADL, and mobility are limited and how they change over time. 3. Extent to which different symptoms relate to ADL disability. 4. Extent to which assistive devices are used and relate to ADL disability. 5. **Determine the extent to which social isolation during the COVID-19 pandemic impacts on ADL function and its recovery** |
| Type of trial | Multi-site prospective cohort study |
| Trial design and methods | - Sample: advanced cancer or respiratory disease (COPD or ILD) - Recruitment sites: hospital lung cancer and respiratory inpatients and outpatient clinics; hospice inpatients, outpatients, or community teams. - Outcome variable: ADL disability (BADL, IADL and mobility) - Explanatory variables: symptoms; assistive devices; **social isolation** - Outcome measures: Modified Barthel Index; Lawton Brody IADL scale; WHO Disability Assessment Scale (WHODAS 2.0); Palliative Outcomes Scale-Symptoms; SARC-F; Frailty measure; **Chronic Disease Self-Efficacy Scales** - Data collection: Baseline self-reported questionnaire **via telephone** and monthly postal survey for 6-months or until death. |
| Health condition(s) or problem(s) studied | Advanced lung cancer or respiratory disease (COPD or ILD) |
| Target sample size | 200 |
| Trial duration per participant: | 6 months or until death/deterioration |
| Main inclusion/exclusion criteria: | Inclusion:   - Patients aged >18 - Advanced lung cancer or respiratory disease (COPD or ILD) defined by advanced disease markers   Exclusion:   - Patients who lack capacity to consent - Patients who lack ability to understand and complete a questionnaire in English - Life expectancy of <1 month, assessed by the person taking consent |
| Statistical methodology and analysis: | - Descriptive statistics will be used to describe the population and changes in ADL disability over time. - Visual graphical analysis (VGA) will be used to categorise individual trajectories and compare patterns. - Regression analysis will be used to test associations between ADL disability and the explanatory variables of symptoms, assistive devices and **social isolation**. - Comparisons will be made across disease groups. |
| **STUDY TIMELINES** | |
| Study Duration/length | 18 months (recruitment over 1 year) |
| Expected Start Date | November 2019 |
| End of Study definition and anticipated date | May 2021 |
| Key Study milestones | **Recruitment opened in March 2020 for 1 year** |

# One Page Summary

This study ‘comparing and contrasting trajectories of disability in activities of daily living (ADL) in advanced lung cancer or respiratory disease’ **during the COVID-19 pandemic’** (DIScOVER) is a prospective cohort study, which aims to compare and contrast trajectories of ADL disability in advanced lung cancer or respiratory disease and their relationship with symptom severity, use of assistive devices and social isolation **during the COVID-19 pandemic** .

This study will compare how ADL disability changes over time between patients with advanced lung cancer or respiratory disease **throughout 2020 during COVID-19 pandemic**. It will recruit patients with a diagnosis of advanced lung cancer or respiratory disease (chronic obstructive pulmonary disease (COPD) or Interstitial lung disease (ILD)) from hospital or hospice inpatient, outpatient or community services and compare the differences in ADL disability and influencing factors, particularly symptom severity, use of assistive devices and **social isolation.** By following people prospectively over time, we will be able to evaluate how ADL disability changes, what influences these changes, who it affects, and whether ADL disability can be modified and how.

This will enable us to better understand how ADL disability affects people with advanced lung cancer or respiratory disease, and where in the trajectory of ADL disability services could potentially be modified and how, in order to improve outcomes for these disease groups. This will inform development and delivery of appropriate interventions and trial design, which will ultimately inform appropriate and timely services addressing ADL disability in advanced disease. **It will also identify the impact social isolation has on a person ability to manage their daily activities and subsequent recovery which may aid future crisis planning.**

*Note on definitions

- ADL disability: the difficulty an individual has in managing everyday activities known as activities of daily living (ADLs), which can be basic (BADL) such as washing or dressing, instrumental (IADL) such as shopping or housework, or mobility-related such as walking or climbing stairs.

# Background and Rationale

- **Epidemiology of advanced cancer or respiratory disease:**

Globally, 9.8 million people died from cancer in 2018 [1], and a further 3 million people died from chronic respiratory disease in 2015 [2]. Living with advanced cancer or respiratory disease brings different challenges. Cancer is often of rapid onset with a severe treatment related symptom burden, whereas respiratory disease is slower to develop but unpredictable in nature [3] and particularly associated with lower social deprivation linked with poor disability-free-life-expectancy [4]. Traditionally palliative care provision differs between the two populations with a strong bias towards cancer [3, 5], as does rehabilitation, with a strong bias towards respiratory disease [6], despite a potential need for both in the two disease groups. Due to an aging population people with a diagnosis of cancer or respiratory disease have an increased likelihood of multi-morbidity [7-10], adding to their complexity and severity of disability [11]. In addition, due to advances in treatment, particularly in lung cancer, people are now living with advanced disease over a longer period-of-time, which may change the illness trajectory in terms of pro-longing symptoms and disability which accompany a chronic condition. This means the needs of people with advanced lung cancer or respiratory disease may be changing, requiring additional strategies for their successful management. Lung cancer is also the most comparable cancer type to respiratory disease which makes for a good comparison of the needs of these two disease groups.

**Disability in activities of daily living in advanced cancer or respiratory disease:**

ADL disability is defined as the difficulty an individual has in managing everyday activities known as activities of daily living (ADLs), which can be basic (BADL) such as washing or dressing, instrumental (IADL) such as shopping or housework, or mobility-related such as walking or climbing stairs. Disability in advanced disease has a specific effect on ADLs, limiting a patient’s independence and quality of life [12]. A recent systematic review identified that trajectories of ADL disability in advanced cancer or respiratory disease can be unchanging, fluctuating or increasing in nature. Increasing ADL disability can be associated with individual factors such as age or gender, illness-related factors such as diagnosis or symptoms, or services such as hospitalization. Towards the end of life disability is often limited by a high burden of symptoms [13], but little is known of the relationship between severity of symptoms and ADL disability specific to lung cancer or respiratory disease and how they compare. Understanding this relationship would enable application of timely and appropriate interventions that modify ADL disability in these populations, such as rehabilitation. Rehabilitation in advanced disease, aims to optimise a patients independence, ability to remain active and improve quality of life during the dying process, by helping people to maintain their optimal levels of physical, sensory, intellectual and social functioning with minimum dependence on others for as long as possible [14-16]^,^. Rehabilitation interventions are very broad making for a difficult comparison. However, there is particularly a lack of study of how assistive devices relate to ADL disability, when, where and for who, which would demonstrate whether ADL disability is modifiable along its trajectory in this population.

- **Need for development work to inform trial design and rehabilitation service provision:**

Rehabilitation interventions towards the end-of-life are complex, comprising multiple component treatments that are adapted to the patient or setting, to target different outcomes. This complexity demands a better understanding of the needs of people receiving rehabilitation, to enable modelling of interventions prior to formal testing and evaluation, in line with the MRC guidelines for developing complex interventions [17]. Development work is particularly important in advanced disease because people present with difficult problems and vary considerably in their level of function, prognosis and reasons for engaging with services [18, 19]. This study will observe trajectories of ADL disability over time, which will enable identification of parameters for rehabilitation, in order to inform development and delivery of an appropriate intervention targeting ADL disability and future trial design. Testing the effectiveness of such an intervention will allow standardization of rehabilitation in advanced disease in order to guide equitable service provision.

- **Adapting study to the COVID-19 Pandemic**

**Coronavirus (COVID-19) was declared a global pandemic by the World Health Organization on 11th March 2020 and as of the 10^th^ April 2020 there have been 60,737 confirmed cases and 7,097 confirmed deaths in the UK [20]. An emergency bill to strengthen the COVID-19 response was put in place on the 17^th^ March 2020 and enforced on the 24^th^ March 2020 [21]. As part of this response, the government enforced social distancing rules on everyone in society and patients with lung cancer or respiratory disease have been advised to stay at home and socially isolate for 12 weeks [22]. Social isolation refers to a complete or near-complete lack of contact with society [23].There are two levels of social isolation imposed on people considered to be at risk of dying from Coronavirus:**

- **Self-isolation: people who are in a high-risk group (e.g. aged over 70, respiratory disease, cancer, diabetes or pregnancy) are advised to stay at home for 12 weeks except for essential errands and avoid contact with others [22].**
- **Shielding: people who are in a very-high risk group including those with severe respiratory disease are strongly advised to stay at home, avoid contact with others including household members and not go out at all for at least 12 weeks from the day they receive a letter from the government which defines them as a vulnerable person (24^th^ March 2020) [24].**

**PPI members have highlighted concerns around reduced professional support and increased demand on informal carers while socially isolating. Pilot participants have added concerns around discrepancies in government support available between high-risk and very-high-risk groups, uncertainty of not knowing how long the situation will last and increasing anxiety around loss of function and ability to cope at home during this period.**

**Social isolation is known to be strongly associated with functional impairments in older people and persons with cancer [23, 25], and is a major contributor of mortality in older adults [26]. If a person is contained to home for a long time, this physical inactivity may cause them to decondition in the same way they would if they had a long stay in hospital. In a study of advanced COPD patients admitted to hospital 50% showed functional decline throughout the six week admission of which only 16.7% recovered functional loss six weeks post discharge [27]. Functional limitation, living alone, and lack of social support are also predictors of emergency attendance and hospital admission in COPD and older people [28-30], which could put increased strain on an already stretched health and social care services during this public health crisis.**

**It is currently unclear what the consequences of enforced social isolation in people with advanced lung cancer or respiratory disease will be on their daily function and the impact decline in function may have on health and social care services during and after the COVID-19 pandemic, whether or not they contract the virus [31]. It is important to understand functional trajectories in the community in the context of COVID-19 and consider the magnitude and long term impact of functional decline in order to help us to understand how this population is affected by the coverinpandemic and plan accordingly for rehabilitation and social care needs.**

# Aims and Objectives

***Aim:***

To compare and contrast trajectories of disability in activities of daily living (ADLs) over time, among adults with advanced lung cancer or respiratory disease (COPD or ILD) **during the COVID-19 pandemic**

**Objectives:**

To describe and compare in people with advanced lung cancer or respiratory disease the following:

1. Trajectories of symptom severity and ADL disability over 6 months.
2. Extent to which different disability items of BADL, IADL, and mobility are limited and how they change over time.
3. Extent to which different symptoms relate to ADL disability.
4. Extent to which assistive devices are used and relate to ADL disability.
5. **Determine the extent to which social isolation during the COVID-19 pandemic impacts on ADL function and its recovery**

**Hypotheses:**

- People with advanced lung cancer develop greater ADL disability over time than people with advanced respiratory disease.
- Symptom severity is positively associated with subsequent ADL disability.
- Use of assistive device is positively associated with increased independence in ADLs.
- **Social isolation is positively associated with increased dependence in ADLs.**

# Methods

**a) Overview of study design:**

This is a multicentre prospective cohort study to observe ADL disability in patients with advanced cancer or respiratory disease. Data will be collected prospectively for 6 months, in a total of 7 monthly assessments including baseline. Figure 1 summarises the study schedule. The data collection booklet will consist of a variety of questionnaires asking patients about disability in ADLS, functioning, symptoms, health care usage and use of assistive devices. These will be collected at all time-points. Demographic and clinical variables will also be collected in the baseline questionnaire only.

*Figure 1: Schedule of prospective data collection*

**Baseline**

**Month 1**

**Month 2**

**Month 3**

**Month 4**

**Month 5**

**Month 6**

**End of 6-month follow-up**

**Consent**

**Self-reported baseline questionnaire**

**Postal follow-up questionnaire**

**Postal follow-up questionnaire**

**Postal follow-up questionnaire**

**Postal follow-up questionnaire**

**Postal follow-up questionnaire**

**Postal follow-up questionnaire**

**b) Study population and sample:**

The study population is patients with advanced cancer or respiratory disease defined as below in section c). Consecutive sampling will be used and include all patients that have been screened eligible and are willing to take part. A convenience sample of patients at local sites will be used for piloting the questionnaire.

**c) Inclusion & exclusion criteria**

Inclusion:

- Patients aged >18
- Advanced lung cancer or respiratory disease as defined by one of the following:
  - *Lung cancer:* Inoperable stage III or IV non-small cell lung cancer
  - *Chronic Obstructive Lung Disease (COPD):* Severe or very severe stages of COPD according to the criteria set by the Global Initiative for Chronic Obstructive Lung Disease (GOLD) [32]: stage III (FEV1/FVC < 70%. 30% ≤ FEV1 < 50% predicted with or without chronic symptoms (cough, sputum production)) and stage IV (FEV1/FVC < 70%. FEV1 < 30% predicted plus chronic respiratory failure)
  - *Interstitial lung disease (ILD):* Carbon monoxide transfer factor (TLCO/DLCO) level of <40% or FVC <50% predicted [33, 34]

Exclusion:

- Patients aged <18
- Patients who lack capacity to consent
- Patients who lack ability to understand and complete a questionnaire in English
- Life expectancy of <1 month as assessed by the person taking consent

**d) Recruitment of Participants**

1. **Setting and identification of participants**

Patients will be recruited in the UK from: hospital medical, respiratory or oncology wards; outpatient lung cancer or respiratory clinics; or hospice/palliative care services, including inpatients, outpatients, community teams, day hospices and rehabilitation services. We will open 5 recruitment sites as follows:

1. King’s College Hospital NHS Foundation Trust, London: lung cancer and COPD
2. Guys’ and St Thomas’ Hospital, London: lung cancer, COPD, and ILD
3. Nottingham University Hospitals NHS Trust: lung cancer
4. St Christopher’s Hospice, London: lung cancer, COPD, and ILD
5. St Michael’s Hospice, East Sussex: lung cancer, COPD, and ILD
6. Macclesfield Hospital, East Cheshire NHS Trust: lung cancer, COPD, and ILD
7. South Tyneside and Sunderland NHS Foundation Trust: lung cancer, COPD, and ILD
8. Medway Foundation Trust, Kent: lung cancer, COPD, and ILD
9. **The British Lung Foundation Charity, National: lung cancer, COPD, and ILD**

The study will start by opening at locally at Kings College Hospital NHS FT to collect pilot data. Following the pilot, the study will formally open for recruitment and the remaining sites will open. In addition, the study will be added to the NIHR portfolio which if needed will enable interested sites with access to this population to volunteer to recruit participants. Each site will recruit approximately 25 participants over the course of 1 year at the rate of approximately 2-3 patients per month per site and followed for six months. The study will therefore be open for 18 months to complete follow-up. Potential participants will be screened and identified by the patients’ direct clinical team at the recruitment sites and asked if they would like to participate in the study. These would be:

- *Inpatient approaches:*
- Identification of patients from hospital/hospice admissions lists
- Identification of patients from ward rounds on suitable inpatient wards
- Identification of patients via ward multidisciplinary meeting discussions
- *Outpatient approaches:*
- Clinicians in oncology and respiratory outpatient clinics at hospital **sites including remote consultations**
- Clinical research nurses where available to attend above clinics to help clinicians screen clinic lists
- Clinicians in hospice outpatients, community services, day hospices and rehabilitation services

In order to understand which of the patients identified by the clinical team as eligible are included, we will record reason for not being approached or reason for decline for non-participants identified by the clinical teams. We will not record any identifiable information for non-participants.

1. **a) Usual Consent Procedure**

Figure 1a outlines the usual consent procedure. Potentially eligible patients will be identified by the clinical team. A member of the participating clinical teams will approach eligible patients and give them a participant information sheet, which details the aim of the study and clearly describes what participation involves. The clinician will ask for verbal consent for the research team to contact the patient, and if this is given will inform the researcher of a potential participant in the study. Patients and their families are given at least 24 hours to read the information before they are contacted by the researcher unless they prefer to discuss the study with a researcher sooner.

The researcher will be informed of any potential participants and will follow up in-person or via telephone or email, as per the patients’ preference. At this point, the researcher will address any questions or concerns and ascertain the patient’s intention to participate or not during this meeting. If they are happy to take part, completion of the consent form and baseline questionnaires will be scheduled at a time and place convenient for the participant. They will be asked to give written informed consent once they have understood the benefits, risks and burdens associated with the study, had all information about the study and are aware that they can withdraw at any time. For patients who are visually impaired or unable to write, there is an option for a witness signature to confirm the patient gives informed consent.

*Figure 1a: Usual consent procedure*

Patient identified by clinical team

Information leaflet given and research team informed

24h

Researcher approaches patient, confirms eligibility, answers any questions and assesses capacity to consent

Written consent is taken and the patient is assigned a study number and formally included in the study

1. **b) Remote Consent during COVID-19 pandemic**

**Figure 2 outlines the remote consent procedure during the COVID-19 pandemic when nonessential patient contact is prohibited. Due to social distancing rules enforced by the government, procedures of consent will move to remote practices and follow HRA guidelines [35], using the process outlined below:**

**- Potentially eligible patients will be identified by one of two new routes:**

1. **An advertisement will be emailed to members of the British lung Foundation (BLF) and to potential participants at recruitment sites already consenting to be contacted by researchers. Interested people are invited to contact the researcher and request an information sheet.**
2. **The clinical team at open recruitment sites, will approach eligible patients, during a routine telephone consultation and introduce the study. The clinician will ask for verbal consent for the research team to contact the patient. If consent is given, they will inform the researcher of a potential participant and email the participants contact details to the researchers NHS email account. The research team will post or email a study pack including a participant information sheet, consent form and the baseline questionnaire and allow at least 24 hours for them to consider participation.**

**- The research team will post or email (as per their preferred option) a study pack including a participant information sheet, consent form and the baseline questionnaire and allow at least 24 hours for them to consider participation.**

**- The researcher will telephone the patient to introduce the study and address any questions or concerns and ascertain the patient’s intention to participate or not during this call.**

**- If the participant is happy to take part, the researcher will verbally consent the participant to be recruited to the study once they have understood the benefits, risks and burdens associated with the study, had all information about the study and are aware that they can withdraw at any time. Verbal consent is permissible as this is not a clinical trial and not considered high-risk to participants [35].**

**- The participant will be asked to return the completed questionnaire in separate stamped addressed envelopes or to the researchers NHS email address. If the participant requires help to complete the questionnaire this will be ascertained at the time of verbal consent and a convenient time to complete the questionnaire over the telephone with the researcher will be arranged. Full consent will be assumed upon receipt of the questionnaire.**

**- The monthly follow-up questionnaires will be delivered either by post or email as per the participants preference.** **RIP status will be checked where possible prior to follow-up. The postal follow-up procedures have not changed.**

***Figure 1b: Remote consent procedure***

**Patient identified and approached by clinical team or BLF**

**Study pack consisting of information leaflet, consent form and baseline questionnaire posted to participant**

**Researcher telephones patient, confirms eligibility, answers any questions and assesses capacity to consent**

**Consent is assumed on receipt of questionnaire, and the patient is assigned a study number and is formally included in the study**

1. **Data collection schedule**

- Study enrolment and baseline assessment

For those participants who meet the inclusion criteria and who consent to participate they will be enrolled in the study by the researcher. Each participant will be provided with an enrolment ID and added to the recruitment log. The researcher will approach the participant to arrange a convenient time to complete the consent form and baseline questionnaire over the telephone which will take approximately 60 minutes to complete. Following consent the researcher will send a standard letter to the participants’ GP informing them of their involvement in the study.

- Follow-up questionnaires and continued consent

There will be six monthly follow-up questionnaires for up to six months from study enrolment. Participants will receive a reminder phone-call a couple of days prior to posting each questionnaire. A couple of days prior to this phone-call clinical teams will inform the researcher of the participants health status, and if they are reported to have died, they will be withdrawn from the study. If the participant is happy to continue in the study, follow-up questionnaires will be posted out to the participants desired address by the researcher at monthly intervals for 6 months from the date of enrolment. There will be no financial incentive for participants to complete follow-up questionnaires. All follow-up questionnaires will take approximately 30-60 minutes to complete. Those who require help to complete the questionnaires can request assistance from the researcher over the telephone or face-to-face depending on the capacity of the recruitment site. A pre-paid envelope will be supplied for returning all questionnaires ideally within 7 days of receipt. If the questionnaire is not returned within the timeframe the participant will receive a telephone call to remind them. Patients will exit the study at 6 months or at the point of death. Information on withdrawal or loss to follow-up will be recorded (e.g. death, deterioration, hospital admission) from the medical notes.

1. **Data collection measures**

The outcome variable of interest is ADL disability consisting of BADL, IADL and mobility. There is no one measure of ADL disability that includes all desired components of BADL, IADL and mobility, therefore individual measures for each are recommended [36]. These are measured using validated outcome measures: Modified Barthel Index (BI) for BADL [37, 38], Lawton Brody IADL Scale (LB) for IADL [39-41] and the mobility domain of the WHO Disability Assessment Schedule 2.0 (WHO DAS mob.) for mobility [42-44]. **The main three explanatory variables of interest are (i) symptoms, (ii) assistive devices, (iii) social isolation measured by the Palliative Outcomes Scale - Symptoms (POS-S) [45] and specific assistive device questions [46], and description of social isolation and self-efficacy using the Chronic Disease Self-Efficacy Scale on Social support respectively.** It is also important to collect all potential confounding variables. All potential variables and data collection measures to be used in prospective data collection are identified in Table 2. Where possible, data will be extracted from clinical notes on enrolment to reduce questionnaire burden.

*Table 2: Data collection measures to be used in prospective data collection*

*2a) Outcome variable – ADL Disability (BADL, IADL, mobility)*

| **Outcome** | **Outcome measure** | **Reason for inclusion** | **Use in analysis** |
| --- | --- | --- | --- |
| Basic activities of daily living (BADL) | Modified Barthel Index (BI) | To understand disability aspects and changes over time in each ADL domain | Primary outcome variable |
| Instrumental activities of daily living (IADL) | Lawton Brody IADL scale (LB) |  | Secondary outcome variable |
| Mobility | Mobility domain of WHODAS 2.0 (WHODAS mob) |  |  |
| Global disability | WHO Disability Assessment Schedule 2.0 (WHO DAS 2.0) | To describe and understand relationship between ADL disability and global disability | Secondary outcome variable |

*2b) Potential explanatory variables*

| **Variable** | **Method of collection** | **Reason for inclusion** | **Use in analysis** |
| --- | --- | --- | --- |
| **Individual factors** |  |  |  |
| Demographic data:   - Age - Gender - Ethnicity - Marital status - Education - Living status - Co-morbidities | List of participant characteristics from participant or medical records  Charlson co-morbidity index | Understand study population and to account for confounding variables | Descriptive – to describe population  Independent variables in analysis |
| Functional performance status | Australian Karnofsky Performance Status (AKPS) | Measure of function | Descriptive – second measure of function which is useful to understand population recruited |
| **Illness-related factors** | |  |  |
| Disease and stage | Diagnosis, date of diagnosis and staging recorded in medical records | Understand study population | Descriptive – to describe population |
| Symptoms | Palliative outcomes scale - Symptoms (POS-S) – 7-day version | To understand relationship between symptoms and ADL disability and change over time | Descriptive – to describe population  Analytic – associations with ADL disability and to compare between groups |

| **Environmental factors** | |  |  |
| --- | --- | --- | --- |
| **Social Isolation** | **Specific questions on COVID-19 and social isolation and the** **Chronic Disease Self-Efficacy scale on social support** | **To understand relationship between social isolation and ADL disability and change over time** | **Descriptive – to describe population**  **Analytic – associations with ADL disability and to compare between groups** |
| Assistive devices | Specific questions on type of assistive device used for different ADL tasks | To understand use of assistive devices and how they relate to ADL disability | Descriptive – to describe use  Analytic – associations with ADL disability and to compare between groups |
| **Service-related factors** |  |  |  |
| Service utilisation | Client service receipt inventory – service receipt section | To understand which services influence ADL disability or are an outcome of ADL disability | Descriptive – to describe use of services  Independent variable  in analysis |
| Place of care / death | Reported by participant or proxy or medical records | To understand impact of ADL disability on place of care or death | Descriptive – to describe location of care or death  Independent variable  in analysis |

**g) Study Procedure**

1. Pilot phase

The questionnaires and data collection methods will be piloted using a brief discussion with the first five consecutively recruited patients following completion of the baseline questionnaire, in order to refine chosen questionnaires and methods. Patients will be asked whether they think the questions work, are understandable and how they would prefer to be contacted, using a semi-structured topic guide. This will allow for sites to test the feasibility of recruiting this patient group and identify any barriers to recruitment. It will also enable the researcher to check the practicality and patients’ understanding of the questionnaire. Any concerns identified with the questionnaire tools will then be quickly relayed to other sites and amendments sought in order to maximise the quality of the data to be collected.

1. Documentation of participation

All participants who consent to participate in the study will be given a copy of the information sheet to keep. The research team will retain the original signed consent form. A copy of the signed consent form will be filed in the patient’s medical notes and they will be offered a signed copy to keep if they wish.

**h) Safety & reporting**

This is an observational study. There will be no intervention or changes to the patient care for the participant if they agree to participate. The steps below will be taken to minimise any distress the participant may experience from completing the survey.

1. Steps to prevent harm

The researcher will make every effort to complete all questionnaires in a private place. The purpose and intent of the study will be explained to participants and the participants will be advised that they are under no obligation to take part. Patients will be made aware that they can withdraw from the study at any time, with no adverse implications for their clinical care.

It is possible that participants may raise issues during the baseline questionnaires which raise clinical concerns or warrant a change in their medical management. Should this be the case, then a member of the research team will gain consent from the participant to discuss matters with the relevant member(s) of the patient’s medical team or their general practitioner, as appropriate. All returned questionnaires will be screened immediately following completion to check their content for any areas of clinical concern. This screening will be done by the researcher at the return address (King’s College London). Additionally, if participants disclose any ideation of self-harm or other risk to themselves or others, then this will be dealt with as an urgent matter for discussion with the PI and a senior member of the treating medical team. Provision will be made to ensure the researchers have PI or senior back up available by phone whenever they are undertaking data collection.

1. Distress protocol

We are aware of the possibility that despite measures to prevent harm, completion of the study questionnaires may be distressing to potential participants. We expect significant distress to be uncommon, since most of the questionnaire deals with routine or day to day issues. Nevertheless, a series of measures will be in place to deal with the any additional distress which may arise in the course of the study as outlined below:

- Study contact telephone number will be made available for participants to contact with any questions or concerns about the study process or in the event of any distress.
- Contact number to be included on all study information and participants to be informed of this during participation.
- Senior clinical staff members will be available to support the researcher and to deal with more complex distress or concerns.

## Withdrawal of participants

In consenting to participate in the survey, participants are consenting to completing a baseline questionnaire with the researcher and 6 follow-up postal surveys.

A participant may withdraw from the study at any time with no effect to their routine care. The decision to withdraw will be included if the participant volunteers that information and will be recorded in the main study database.

**i) Data handling and management**

All personal data will be managed according to the principles established in the Data Protection Act 2018. All of the researchers will undertake and update GCP training, and current research governance processes will be followed. Completed demographic forms, questionnaires and interview transcripts will be anonymised using a unique study identification number and contain no patient identifiable data. The participant identification number and linkage with the participant’s name will only occur on the consent form and participant log. The participant log will be held in a password protected Excel Spread sheet, stored on an encrypted memory stick at KCL in a locked filing cabinet, and backed up on an NHS computer in a separate location. Data will be transferred via NHS or other secure email account. Questionnaires, demographics forms and transcripts will be stored separately to the consent forms, each in a separate locked cabinet.

ALL personal data held by the research team will be stored for seven years after the study has ended. This is to allow enough time for clarification and validation following reporting and publications. Data will be stored in locked cabinets in a locked office in the Department of Palliative Care, Policy & Rehabilitation in accordance with the requirements of the Data Protection Act 2018 [47] and local Data Management Guidelines.

Data quality will be monitored throughout the study at the local sites, and centrally monitored at the lead site. All data will be de-identified before being transferred to the lead site (King’s College London).

**j) Statistical Analysis**

1. ***Proposed sample size***

The sample size of 200 participants, 100 advanced lung cancer and 100 advanced respiratory disease and/or an allocated proxy (as outlined in consent section above) will be recruited for the prospective questionnaire. This is not a trial, so in the quantitative data we are not estimating a sample size sufficient to determine a difference between one intervention and another. The basis of this sample size estimate is based on a combination of the following factors:

- *Precision:*

The changing trajectories of ADL disability in advanced lung cancer or respiratory disease are not known, therefore descriptive analysis will provide useful new information. Thurs we will be aiming to estimate these with a level of precision and to describe them over time. Based on assumed prevalence of ADL disability in this patient population to be around 50% [2, 12], a sample size of 150 would achieve a precision of 8% in the estimation of prevalence of ADL disability characteristics.

- *Power:*

Comparative analysis will be used to investigate changes over time between the two disease groups (lung cancer and respiratory disease). A recent study within the department investigating trajectories of need, experience and priorities over time in older adults with serious illness (IARE2), collected ADL data using the Modified Barthel index (BI) at three time-points over 6 months. IARE2 has a similar advanced illness population to this study and included patients with a diagnosis of cancer or respiratory disease. Therefore in order to work out our sample size it was possible to use cases of cancer (solid tumours) and COPD that had BI scores at baseline and 6 months from this data to calculate an effect size using the following formula by Cohen 1988 [48]:

d (effect size) = M1 *(mean of group 1)* - M2 *(mean of group 2)*

√ (SD1² + SD2²) / 2

A sample size was then calculated using G-power software. Based on a difference between two independent groups and an effect size of 0.45 on the BI (primary outcome) at the primary end point of 6 months with power of 80% at a 5% significance level, a sample size of 158 would be required.

*Figure 3: Sample size calculation*


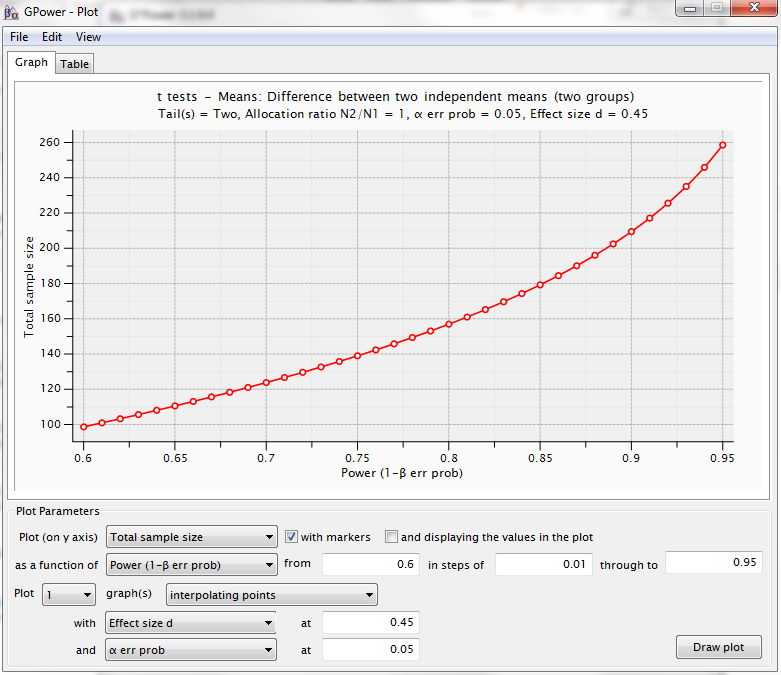


- *Adjustment for co-variates in regression analysis:*

We will be using regression analysis to test the association between the outcome variable of ADL disability and the explanatory variable (co-variate). As a rule of thumb in regression analysis there should be ten cases for each co-variate. A sample size of 200 allows for up to 20 co-variates in the analysis. The co-variates that need to be accounted for in this study are age, gender, ethnicity, martial-status, education level, living status, co-morbidities, performance status, diagnosis, symptom severity, cognitive function, assistive devices, service utilization, place of care, place of death, and mortality. Therefore, a minimum sample size of 160 is required.

- *Attrition:*

An attrition rate of 20% over 6 months was estimated in this study population. This is based on a local study in the advanced disease population that experienced 36% attrition over 12 months [49]. This means by recruiting 200 participants by the end of the study our sample size will be 160. Therefore, our estimated sample size will be sufficient to account for a precision of 8%, power of 80% at a 5% significance level, and adjustment of the required co-variates in the analysis.

1. ***Principles of analysis and data usage:***

Questionnaires will be checked after completion for any concerning clinical features. All questionnaire data will be entered into a standardised spreadsheet using Microsoft Excel, which will be standardised across sites. Data will be checked for missing values. Statistical software will be used to support descriptive and comparative analysis. This will be done by the researcher.

1. ***Specific analysis plan***

Assessment of disability

ADL disability is the primary outcome and will be measured using three measures: Modified Barthel Index (BI) for BADL (main primary), Lawton Brody IADL Scale (LB) for IADL and the World Health Organisation Disability Assessment Schedule 2.0 (WHOSDAS 2.0) mobility domain (WHODAS-mob) for mobility. Disability is defined on each of these measures as follows:

BI: A 10-item categorical measure, where each item has a range of two to four responses rated on a 0-4 scale, ranging from dependent/unable, to minor help, major help, or independent, depending on the activity. Therefore a lower score indicates greater disability. A summary score ranges from 0-20 where a score <15 represents moderate disability and a score <10 represents severe disability [37]. Changes of more than 2 points in total score reflect a probable genuine change in ability to perform ADLs [37].

LB: An 8-item categorical measure, where each item has a range of three to five responses ranging from fully independent to fully dependent. Each response is scored 0 if independent or 1 for anything other than independent. A summary score ranges from 0 (low function, dependent) to 8 (high function, independent), therefore a lower score indicates greater disability [41, 50]. A change of 1 or more in total score indicates a change in ADL disability.

WHODAS-mob: A 5-item categorical measure, where the patient rates difficulty in each task on a 5-point scale from none (1) to extreme or unable (5). A simple summary score totalling the scores of all five items ranges from 5-25 where the lowest score indicates no disability and the highest score indicates extreme disability [43, 44]. A score of 5 or more indicates moderate disability. A more comprehensive scoring method will also be used (see WHODAS 2.0 below).

WHODAS 2.0: All domain scores rated as above (see WHODAS-mob) on the WHODAS 2.0 are combined to give a global disability score, with higher scores indicating higher levels of disability [44]. To enable easy comparison each domain score can be transformed to a 0-100 scale using the complex WHODAS method of scoring [51]. Global disability scores are categorized according to ICF severity ranges (no problem, 0-4; mild disability, 5-24; moderate disability, 25-49, severe/extreme disability, 50-100). Based on the WHODAS ICF, ‘disability’ is indicated by a disability score of 25 or higher.

The individual items measured in each ADL tool (BI, LB and WHODAS-mob) are listed in Table 3. For individual items in line with the scale metric a change in disability on any measure is identified by a change of 1 point or more in any direction (improvement or decline).

*Table 3: Individual items of each outcome measure for use in analysis*

| **Modified Barthel Index** | **Lawton-Brody IADL Scale** | **WHODAS-Mob** | **POS-S** | **Assistive devices** |
| --- | --- | --- | --- | --- |
| Bowels | Ability to use telephone | Standing for long periods (>30mins) | Pain | Special utensils/dishes to help eating |
| Bladder | Shopping | Standing up from sitting down | Shortness of breath | Equipment to help getting in or out of bed |
| Grooming | Food preparation | Moving around inside the home | Weakness or lack of energy | Equipment to help getting around inside |
| Toilet use | Housekeeping | Getting out of the home | Nausea | Special clothing or use equipment to get dressed |
| Feeding | Laundry | Walking long distances (1km) | Vomiting | Equipment to help bathe |
| Transfer (bed to chair and back) | Mode of transportation |  | Poor appetite | Equipment to help use the toilet |
| Mobility | Responsibility of own medication |  | Constipation | Equipment to help getting around outside |
| Dressing | Ability to handle finances |  | Mouth Problems | Equipment to help use the stairs or steps |
| Stairs |  |  | Drowsiness | Equipment to help with domestic tasks |
| Bathing |  |  | Immobility | Anything else (e.g. transport adaptations or communication aids) |

Assessment of symptom severity

Symptom severity will be measured using the POS-S which is a 10-item categorical measure listing ten symptoms (Table 3). The patient rates the severity of each symptom on a 5-point scale from not at all (0) to overwhelmingly affected (4). A summary score ranges from 0-20, where a higher score indicates greater severity of overall symptoms. According to the scale a change in individual symptom severity is identified by a change of 1 point or more in any direction (improving or worsening).

Assessment of assistive devices

This is measured using a list of ten questions about assistive devices to help with several ADL tasks (Table 3), which have been used in previous surveys [46]. Patients answer yes or no to the use of equipment for each task, followed by a question asking them to specify what equipment they use. This can be measured on a binary scale (yes: 1, no: 0), making a combined summary score of 0-7 where a higher total score indicates a higher use of assistive devices and greater disability.

**Assessment of Social Isolation and self-efficacy**

**Questions will be added around COVID-19 and social isolation including information on how the participant’s daily activity and support changed while self-isolating or shielding. Strategies used to manage physical well-being will also be collected. The ‘receiving social support’ scale from the validated Chronic Disease Self-Efficacy Scales will be used to measure confidence in managing receiving social support in society’ [52, 53]. This scale contains 4 questions which are scored on a numerical rating scale of 1 to 10 where 1 is ‘not at all confident’ and 10 is ‘totally confident’. The total score is the mean of the 4 items. A higher score indicates higher self-efficacy in that scale.**

Descriptive analysis:

The data from patient questionnaires will initially be summarised using descriptive statistics to describe the population in detail and make comparisons between the two disease groups. This will include demographic characteristics, baseline performance status, symptom severity, assistive device use, **social isolation,** service utilization, and place or care or/and death (Table 2). The significance of variations will be determined using Χ^2^ tests or Fishers Exact Test when required for categorical data, Mann Whitney U tests for ordinal data and t-tests/ANOVA for continuous data. A significance value of p < 0.05 will be used.

- Analysis by objectives:

To describe and compare in people with advanced lung cancer or respiratory disease the following:

1. *Trajectories of symptom severity and ADL disability over 6 months.*

*Summary trajectories:*

We will describe changes in ADL disability (BADL, IADL, mobility) using total scores on each of the three measures (BI, LB and WHODAS-mob) and for symptom severity (POS-S total score) over time using repeated measures from point of study entry (forward trajectory) and from point of death (backwards trajectory). Forward trajectories are useful to understand clinical implications of ADL disability and symptom severity and backward trajectories allow understanding of how these change prior to death. Trajectories of ADL disability and symptom severity will be determined separately for lung cancer and respiratory disease using summary statistics with means and 95% confidence intervals at each time-point and will be presented graphically.

*Individual trajectories:*

Individual trajectories using the primary outcome of ADL disability (BADL, IADL, mobility) using visual graphical analysis (VGA) [54] which allows patterns to emerge for visual inspection of each individual report which have completed data from repeated measures at three or more time-points. These trajectories will be plotted separately for lung cancer and respiratory disease to identify variances in common patterns and develop categories of trajectories of ADL disability (e.g. increasing, decreasing, stable or fluctuating). The clinical and demographic characteristics of each trajectory group will be described and compared using Χ^2^ tests or Fishers Exact Test for categorical variables and appropriate non-parametric analysis of variance for continuous variables (t-test, Mann-Whitney U, or Kruskal-Wallis test). If appropriate latent growth curve modelling may be used.

1. *Extent to which different disability items of BADL, IADL, and mobility are limited and how they change over time.*

The prevalence disability in each individual ADL item (Table 3) for all three measures (BI, LB and WHODAS-mob) will be calculated by dividing the number of participants with disability in that item in each specific month by the total of completed questionnaires that month, which will be tabulated. Disability is classed in the individual items of each measure as:

- BI: needs help/dependent/unable (scores vary per item)
- LB: score of 1 point or more
- WHODAS-mob: score of 1 point or more

Change in monthly prevalence of each item can be plotted over time using summary statistics with means and 95% confidence intervals at each time-point and presented graphically. Lung cancer and respiratory disease will be analysed separately in order to make comparisons between the two disease groups.

1. *Extent to which different symptoms limit ADL disability.*

Symptoms recorded using POS-S will be described using descriptive statistics and tabulated. We will undertake regression analysis using overall change in each ADL measure (BI, LB and WHODAS-mob) as the outcome variable to test associations with change in each individual symptom score on the POS-S over time using summary statistics with means and 95% confidence intervals at each time-point. Lung cancer and respiratory disease will be analysed separately in order to make comparisons between the two disease groups.

1. *Extent to which assistive devices are used and relate to ADL disability.*

The types of assistive devices (Table 3) used by participants will be described using descriptive statistics. The prevalence of assistive devices will be calculated by dividing the number of participants using any assistive device in a specific month by the total of completed questionnaires that month and change can be plotted over time using summary statistics with means and 95% confidence intervals at each time-point. This can also be done for each individual assistive device to compare popularity/availability. We will undertake regression analysis using overall change in each ADL measure to test associations with use of assistive devices as measured by change in prevalence over time. Lung cancer and respiratory disease will be analysed separately in order to make comparisons between the two disease groups.

1. **Determine the extent to which social isolation during the COVID-19 pandemic impacts on ADL function and its recovery**

**The level of social isolation, length of time spent socially isolating, effect on physical activity and support with daily living, and self-efficacy score in the ‘chores’, ‘receiving social support’ and ‘participation in society’ scales, will be described using descriptive statistics and presented as frequencies, means or medians as appropriate. We will undertake regression analysis using overall change in each ADL measure to test associations with level of social isolation and change in self-efficacy score over time. Lung cancer and respiratory disease will be analysed separately in order to make comparisons between the two disease groups.**

- Adjustments in analysis

Adjustments will be made in the analysis for multiple testing, missing data and confounding variables as follows:

Multiple testing: To adjust for multiple comparisons in this analysis a multiple testing correction such as the Bonforroni, Holm, Hochberg or Hommel adjustment can be used [55]. As a compromise the significance level will be set to a p-value of 0.01.

Missing data: Analysis will test the pattern of missing data and depending on the nature of missingness (at random, not at random, completely not at random) will use and contrast findings using sensitivity analysis [56].

Confounding variables: Adjustments will be made in the multivariate analysis for covariates as appropriate (e.g. time, age, gender, ethnicity, martial-status, education level, living status, co-morbidities, performance status, diagnosis, symptom severity, cognitive function, assistive devices, service utilization, place of care, **social isolation, self-efficacy**).

**k) Patient and public involvement (PPI)**

The public engagement forum at the Cicely Saunders Institute will be utilized to engage patients and members of the public in the planning of the study and screening of all study documents to ensure appropriateness. They will be updated on the progress of the study and involved in the dissemination of findings.

**l) Ethical and regulatory approval**

This study will be conducted in line with principles of research ethics as outlined in the declaration of Helsinki and Good Clinical Practice (GCP) guidance. This protocol and study documents will be submitted to the Health Research Authority for approval.

**m) Dissemination**

Knowledge will be presented through the project (within 3 years) to:

- researchers and clinicians within the project group
- clinicians involved with the research at recruitment sites as findings emerge
- participants/caregivers who express interest
- at Institutional open seminar programmes

Learning will be shared (within 5 years):

- in peer reviewed publications in high impact journals
- at national (e.g. CSP Congress, SRR) and international conferences (e.g. European Association of Palliative Care)
- with clinicians at speciality study events (e.g. ACPOPC (Association of chartered physiotherapists in oncology and palliative care) seminars)
- with students on KCL’s longstanding physiotherapy and Palliative Care programmes
- via our department website and other online channels including YouTube and Twitter

**n) Funding and costings**

Funder: NIHR grant to the value of £565,413, covering:

- 3-year research assistant salary (£32,548 per annum)
- Printing and posting of questionnaires with pre-paid return envelopes (£5000)

**o) Revised Project timeline**

| 2019 | | | | 2020 | | | | | | | | | | | | 2021 | | | | | | | | | |
| --- | --- | --- | --- | --- | --- | --- | --- | --- | --- | --- | --- | --- | --- | --- | --- | --- | --- | --- | --- | --- | --- | --- | --- | --- | --- |
| Sept | Oct | Nov | Dec | Jan | Feb | Mar | April | May | June | July | Aug | Sept | Oct | Nov | Dec | Jan | Feb | Mar | April | May | June | July | Aug | Sept | Oct |
| Ethics | | | | |  |  |  |  |  |  |  |  |  |  |  |  |  |  |  |  |  |  |  |  |  |
|  |  |  |  |  | Site set up | | | |  |  |  |  |  |  |  |  |  |  |  |  |  |  |  |  |  |
|  |  |  |  |  |  | Pilot | |  |  |  |  |  |  |  |  |  |  |  |  |  |  |  |  |  |  |
|  |  |  |  |  |  |  |  | Recruitment | | | | | | | |  |  |  |  |  |  |  |  |  |  |
|  |  |  |  |  |  |  |  | Prospective follow-up | | | | | | | | | | | | | |  |  |  |  |
|  |  |  |  |  |  |  |  |  |  |  |  |  |  |  |  |  |  |  | Data cleaning and Analysis | | | | | |  |
|  |  |  |  |  |  |  |  |  |  |  |  |  |  |  |  |  |  |  |  |  |  |  | Write up | | |
|  |  |  |  |  |  |  |  |  |  |  |  |  |  |  |  |  |  |  |  |  |  |  |  | Paper | |

# References

1. UK, C.R. *Cancer Incidence Statistics*. 2013; Available from: <http://www.cancerresearch.org/health-professional/worldwide-cancer-statistics#heading-One>.

2. al, S.J.e., *Global, regional, and national deaths, prevalence, disability-adjusted life years, and years lived with disability for chronic obstructive pulmonary disease and asthma, 1990–2015: a systematic analysis for the Global Burden of Disease Study 2015.* The Lancet Respiratory Medicine, 2017. **5**(9).

3. Murtagh, F.E., M. Preston, and I. Higginson, *Patterns of dying: palliative care for non-malignant disease.* Clin Med (Lond), 2004. **4**(1): p. 39-44.

4. Wohland, P., et al., *Drivers of inequality in disability-free expectancy at birth and age 85 across space and time in Great Britain.* J Epidemiol Community Health, 2014. **68**(9): p. 826-33.

5. Currow, D.C., et al., *The need to research refractory breathlessness.* Eur Respir J, 2016. **47**(1): p. 342-3.

6. Rugbjerg, M., et al., *Effectiveness of pulmonary rehabilitation in COPD with mild symptoms: a systematic review with meta-analyses.* Int J Chron Obstruct Pulmon Dis, 2015. **10**: p. 791-801.

7. Barnett, K., et al., *Epidemiology of multimorbidity and implications for health care, research, and medical education: a cross-sectional study.* Lancet, 2012. **380**(9836): p. 37-43.

8. Lunney, J.R., et al., *Patterns of functional decline at the end of life.* Jama, 2003. **289**(18): p. 2387-92.

9. Javier, N.S. and M.L. Montagnini, *Rehabilitation of the hospice and palliative care patient.* J Palliat Med, 2011. **14**(5): p. 638-48.

10. Chaudhry, S.I., et al., *Restricting symptoms in the last year of life: a prospective cohort study.* JAMA Intern Med, 2013. **173**(16): p. 1534-40.

11. Murray, S.A., et al., *Illness trajectories and palliative care.* Bmj, 2005. **330**(7498): p. 1007-11.

12. Neo, J., et al., *Disability in activities of daily living among adults with cancer: A systematic review and meta-analysis.* Cancer Treat Rev, 2017. **61**: p. 94-106.

13. Gill, T.M., et al., *Distressing Symptoms, Disability, and Hospice Services at the End of Life: Prospective Cohort Study.* J Am Geriatr Soc, 2017.

14. Kasven-Gonzalez, N., R. Souverain, and S. Miale, *Improving quality of life through rehabilitation in palliative care: case report.* Palliat Support Care, 2010. **8**(3): p. 359-69.

15. Kanach, F.A., L.M. Brown, and R.R. Campbell, *The role of rehabilitation in palliative care services.* Am J Phys Med Rehabil, 2014. **93**(4): p. 342-5.

16. Dietz, J.H., Jr., *Rehabilitation of the cancer patient.* Med Clin North Am, 1969. **53**(3): p. 607-24.

17. Craig, P., et al., *Developing and evaluating complex interventions: the new Medical Research Council guidance.* Bmj, 2008. **337**: p. a1655.

18. Santiago-Palma, J. and R. Payne, *Palliative care and rehabilitation.* Cancer, 2001. **92**(4 Suppl): p. 1049-52.

19. Schleinich, M.A., et al., *Palliative care rehabilitation survey: a pilot study of patients' priorities for rehabilitation goals.* Palliat Med, 2008. **22**(7): p. 822-30.

20. Organisation, W.H. *COVID-19 Health System Response Monitor - United Kingdom*. [cited 2020 10.04.20]; Available from: <https://www.covid19healthsystem.org/countries/unitedkingdom/countrypage.aspx>.

21. Department, o.H. &, and S. Care. *What the Coronavirus Bill will do*. 2020 [cited 2020 10.04.20]; Available from: <https://www.gov.uk/government/publications/coronavirus-bill-what-it-will-do/what-the-coronavirus-bill-will-do>.

22. Department, o.H. &, and S. Care. *COVID-19: Guidance on social distancing for everyone in the UK*. 2020 [cited 2020 10.04.20]; Available from: <https://www.gov.uk/government/publications/covid-19-guidance-on-social-distancing-and-for-vulnerable-people/guidance-on-social-distancing-for-everyone-in-the-uk-and-protecting-older-people-and-vulnerable-adults>.

23. Perissinotto, C., et al., *A Practical Approach to Assessing and Mitigating Loneliness and Isolation in Older Adults.* J Am Geriatr Soc, 2019. **67**(4): p. 657-662.

24. Kmietowicz, Z., *Covid-19: Highest risk patients are asked to stay at*

*home for 12 weeks.* The BMJ, 2020. **368:m1170**.

25. Deckx, L., M. van den Akker, and F. Buntinx, *Risk factors for loneliness in patients with cancer: a systematic literature review and meta-analysis.* Eur J Oncol Nurs, 2014. **18**(5): p. 466-77.

26. Holt-Lunstad, J., et al., *Loneliness and social isolation as risk factors for mortality: a meta-analytic review.* Perspect Psychol Sci, 2015. **10**(2): p. 227-37.

27. Medina-Mirapeix, F., et al., *Patterns, Trajectories, and Predictors of Functional Decline after Hospitalization for Acute Exacerbations in Men with Moderate to Severe Chronic Obstructive Pulmonary Disease: A Longitudinal Study.* PLoS One, 2016. **11**(6): p. e0157377.

28. Garcia-Perez, L., et al., *Risk factors for hospital readmissions in elderly patients: a systematic review.* Qjm, 2011. **104**(8): p. 639-51.

29. Thomas, J.M., L.M. Cooney, Jr., and T.R. Fried, *Systematic review: Health-related characteristics of elderly hospitalized adults and nursing home residents associated with short-term mortality.* J Am Geriatr Soc, 2013. **61**(6): p. 902-11.

30. Aminzadeh, F. and W.B. Dalziel, *Older adults in the emergency department: a systematic review of patterns of use, adverse outcomes, and effectiveness of interventions.* Ann Emerg Med, 2002. **39**(3): p. 238-47.

31. Spruit, M., *COVID-19 and Rehabilitation*. 2020, European Respiratory Society.

32. Disease, G.I.f.C.O.L., *Pocket Guide to COPD Diagnosis, Management and Prevention: A Guide for Health Professionals*. 2018.

33. Caminati, A. and S. Harari, *IPF: New insight in diagnosis and prognosis.* Respir Med, 2010. **104 Suppl 1**: p. S2-10.

34. Bradley, B., et al., *Interstitial lung disease guideline: the British Thoracic Society in collaboration with the Thoracic Society of Australia and New Zealand and the Irish Thoracic Society.* Thorax, 2008. **63 Suppl 5**: p. v1-58.

35. Authority, T.H.R., *Joint statement on seeking consent by electronic methods*. 2018.

36. Jagger C, M.R., King D, Comas-Herrera A, Grundy E, Stuchbury R, Morciano M, Hancock R et al, *Calibrating disability measures across British National Surveys*. 2009, Department of Work and Pensions.

37. Collin, C., et al., *The Barthel ADL Index: a reliability study.* Int Disabil Stud, 1988. **10**(2): p. 61-3.

38. Wade, D.T. and C. Collin, *The Barthel ADL Index: a standard measure of physical disability?* Int Disabil Stud, 1988. **10**(2): p. 64-7.

39. Janaudis-Ferreira, T., et al., *Measurement of activities of daily living in patients with COPD: a systematic review.* Chest, 2014. **145**(2): p. 253-271.

40. Wales, K., et al., *Functional Assessments Used by Occupational Therapists with Older Adults at Risk of Activity and Participation Limitations: A Systematic Review.* PLoS One, 2016. **11**(2): p. e0147980.

41. Lawton, M.P. and E.M. Brody, *Assessment of older people: self-maintaining and instrumental activities of daily living.* Gerontologist, 1969. **9**(3): p. 179-86.

42. Yang, M., X. Ding, and B. Dong, *The measurement of disability in the elderly: a systematic review of self-reported questionnaires.* J Am Med Dir Assoc, 2014. **15**(2): p. 150.e1-9.

43. Ustun TB, K.N., chatterji S, Rehm J., *Measuring Health and Disability: Manual for WHO Disability Assessment Schedule (WHODAS 2.0)*. 2010.

44. Ustun, T.B., et al., *Developing the World Health Organization Disability Assessment Schedule 2.0.* Bull World Health Organ, 2010. **88**(11): p. 815-23.

45. Hearn, J. and I.J. Higginson, *Development and validation of a core outcome measure for palliative care: the palliative care outcome scale. Palliative Care Core Audit Project Advisory Group.* Qual Health Care, 1999. **8**(4): p. 219-27.

46. Cornman, J.C., V.A. Freedman, and E.M. Agree, *Measurement of assistive device use: implications for estimates of device use and disability in late life.* Gerontologist, 2005. **45**(3): p. 347-58.

47. Parliament, B., *Data protection act of 2018*. 2018.

48. J, C., *Statistical power analysis for behavioural sciences*. 1988, Hillsdale: Lawrence Earlbaum Associates.

49. Higginson, I.J., et al., *Symptoms and quality of life in late stage Parkinson syndromes: a longitudinal community study of predictive factors.* PLoS One, 2012. **7**(11): p. e46327.

50. Vittengl, J.R., et al., *Comparative validity of seven scoring systems for the instrumental activities of daily living scale in rural elders.* Aging Ment Health, 2006. **10**(1): p. 40-7.

51. Organization, W.H. *WHO Disability Assess*

*ment*

*Schedule 2.0 (WHODAS 2.0)*. [cited 2019 Sept 2019]; Available from: <https://www.who.int/classifications/icf/more_whodas/en/>.

52. centre, S.m.r. *Chronic Disease Self-Efficacy Scales*. 2020 [cited 2020 10.04.20]; Available from: <https://www.selfmanagementresource.com/docs/pdfs/English_-_chronic_disease_self-efficacy_scales_32.pdf>.

53. Lorig, K., et al., *Development and evaluation of a scale to measure perceived self-efficacy in people with arthritis.* Arthritis Rheum, 1989. **32**(1): p. 37-44.

54. Brown, C.G., et al., *Visual graphical analysis: a technique to investigate symptom trajectories over time.* Nurs Res, 2007. **56**(3): p. 195-201.

55. Chen, S.Y., Z. Feng, and X. Yi, *A general introduction to adjustment for multiple comparisons.* J Thorac Dis, 2017. **9**(6): p. 1725-1729.

56. Fielding, S., et al., *Methods for handling missing data in palliative care research.* Palliat Med, 2006. **20**(8): p. 791-8.
