# Supplementary material for: Predictors of increasing disability in activities of daily living among people with advanced respiratory disease: a multi-site prospective cohort study, England UK
Source: Disabil Rehabil. 2023 Dec 10;46(20):4735–44. doi: 10.1080/09638288.2023.2288673 (PMC11441397; doi:10.1080/09638288.2023.2288673)
Supplement: Supplemental Material [file IDRE_A_2288673_SM7471.zip › Fettes Predictors paper Supplementary material C.docx]

**Supplementary Material C**

Table S1: Uni-variable associations with an increasing disability trajectory in basic activities of daily living (BADL)

| **Participant characteristics and outcomes at baseline** | **Univariable associations (n=97)** | | | **Multivariable associations (n=97)** | | |
| --- | --- | --- | --- | --- | --- | --- |
|  | **odds ratio** | **95% CI** | **p-value** | **odds ratio** | **95% CI** | **P-value** |
| - **Health-related factors** |  |  |  |  |  |  |
| NSCLC | 0.28 | 0.11 - 0.72 | **0.009** | 0.82 | 0.72 – 9.38 | 0.87 |
| Stage IV | 0.98 | 0.38 – 2.49 | 0.96 | - | - | - |
| Charlson comorbidity Index score | 0.85 | 0.75 - 0.98 | **0.01** | 0.9 | 0.72 – 1.11 | 0.33 |
| - **Body Functions and Structures** |  |  |  |  |  |  |
| Australian Karnofsky Performance Status | 0.96 | 0.92 - 0.99 | 0.02 | - | - | - |
| Symptom severity (Palliative care Outcome Scale-symptoms) | 1.1 | 1.02 - 1.19 | **0.01** | 1.05 | 0.93 - 1.18 | 0.72 |
| *Pain* | 1.03 | 0.64 - 1.63 | 0.91 | - | - | - |
| *Shortness of breath* | 1.62 | 1.06 - 2.49 | 0.03 | - | - | - |
| *Weakness or lack of energy* | 1.41 | 0.90 - 2.19 | 0.13 | - | - | - |
| *Nausea* | 1.24 | 0.71 - 2.15 | 0.44 | - | - | - |
| *Vomiting* | 2.19 | 0.81 - 5.92 | 0.12 | - | - | - |
| *Poor appetite* | 1.69 | 1.02 - 2.82 | 0.04 | - | - | - |
| *Constipation* | 1.99 | 1.09 - 3.64 | 0.03 | - | - | - |
| *Mouth problems* | 1.17 | 0.64 - 2.12 | 0.61 | - | - | - |
| *Drowsiness* | 1.27 | 0.83 - 1.93 | 0.27 | - | - | - |
| *Immobility* | 2.92 | 1.84 - 4.66 | **<0.001** | - | - | - |
| Receiving cancer treatment | 0.29 | 0.11 - 0.77 | **0.01** | 6.57 | 0.54-79.83 | 0.14 |
| On oxygen therapy | 3.15 | 1.07 - 9.27 | 0.04 | - | - | - |
| - **Activity and participation** |  |  |  |  |  |  |
| Total Barthel Index score (basic ADLs) | 0.74 | 0.59 - 0.92 | **0.007** | 1.06 | 0.72-1.55 | 0.77 |
| Lawton Brody IADL score (instrumental ADLs) | 0.64 | 0.49 - 0.84 | **0.001** | 0.92 | 0.59-1.47 | 0.75 |
| WHODAS Summary score | 1.04 | 1.01 - 1.06 | **0.008** | - | - | - |
| Cognition | 1.11 | 0.97 - 1.27 | 0.12 | - | - | - |
| Mobility | 1.25 | 1.12 - 1.39 | **<0.001** | 1.13 | 0.96 - 1.33 | 0.15 |
| Self-Care | 1.18 | 1.02 - 1.37 | 0.03 | - | - | - |
| Getting along with people | 1.04 | 0.92 - 1.16 | 0.55 | - | - | - |
| Household activities | 1.17 | 1.06 - 1.29 | **0.001** | - | - | - |
| Societal participation | 1.06 | 0.97 - 1.15 | 0.2 | - | - | - |
| - **Personal Factors** |  |  |  |  |  |  |
| Age | 1.07 | 1.01 - 1.14 | **0.01** | 1.07 | 0.99 - 1.15 | 0.09 |
| Female | 0.8 | 0.30 – 2.12 | 0.66 | 0.89 | 0.26 – 3.08 | 0.86 |
| White British | 0.81 | 0.14 - 4.47 | 0.81 | - | - | - |
| Education above secondary school | 0.73 | 0.29 – 1.86 | 0.5 | - | - | - |
| CDSE: Confidence to receive help | 1.1 | 0.74 – 1.63 | 0.64 | - | - | - |
| - **Environmental factors** |  |  |  |  |  |  |
| Lives alone | 1.39 | 0.53 – 3.65 | 0.5 | 1.1 | 0.27- 4.49 | 0.9 |
| Property with stairs | 0.56 | 0.2 – 1.55 | 0.26 | - | - | - |
| Months spent in physical and social isolation | 1.21 | 1.04-1.43 | 0.02 | - | - | - |
| Receiving community (or hospice) palliative care | 5.33 | 1.87- 15.14 | **0.002** | 3.05 | 0.54 – 17.21 | 0.21 |
| Total number of ADL devices | 1.49 | 1.17 - 1.90 | **0.001** | 1.34 | 0.89 – 3.02 | 0.17 |
| Reduced physical activity indoors | 0.87 | 0.34 – 2.2 | 0.77 | - | - | - |
| Reduced physical activity outdoors | 0.87 | 0.34 – 2.23 | 0.77 | - | - | - |
| **Constant (Increasing disability trajectory)** | **-** | **-** | **-** | **0.00009** | **2.75e-09 – 3.02** | **0.08** |

Table S2: Uni-variable associations with an increasing disability trajectory in instrumental ADLs (IADL)

| **Participant characteristics and outcomes at baseline** | **Univariable associations (n=82)** | | | **Multivariable associations (n=82)** | | |
| --- | --- | --- | --- | --- | --- | --- |
|  | **odds ratio** | **95% CI** | **P-value** | **odds ratio** | **95% CI** | **P-value** |
| - **Health-related factors** |  |  |  |  |  |  |
| *NSCLC* | 0.48 | 0.18 - 1.23 | 0.13 | 6.02 | 1.01 – 35.84 | 0.05 |
| Stage IV | 0.73 | 0.29 – 1.79 | 0.49 | - | - | - |
| Charlson comorbidity Index score | 0.93 | 0.83 - 1.04 | 0.18 | - | - | - |
| - **Body Functions and Structures** |  |  |  |  |  |  |
| Australian Karnofsky Performance Status | 0.92 | 0.88 - 0.96 | **<0.001** | - | - | - |
| Symptom severity (Palliative care Outcome Scale-symptoms) | 1.15 | 1.05 - 1.27 | **0.002** | 1.03 | 0.89 - 1.2 | 0.69 |
| *Pain* | 1.9 | 1.23 - 3.25 | **0.005** | - | - | - |
| *Shortness of breath* | 1.59 | 1.08 - 2.3 | 0.02 | - | - | - |
| *Weakness or lack of energy* | 1.53 | 1.03 - 2.29 | 0.03 | - | - | - |
| *Nausea* | 2.38 | 1.06 - 5.32 | 0.04 | - | - | - |
| *Vomiting* | - | - | - | - | - | - |
| *Poor appetite* | 2.38 | 1.28 -4.39 | **0.006** | - | - | - |
| *Constipation* | 1.82 | 1 - 3.46 | 0.07 | - | - | - |
| *Mouth problems* | 1.56 | 0.82 - 2.96 | 0.17 | - | - | - |
| *Drowsiness* | 1.16 | 0.77 - 1.76 | 0.48 | - | - | - |
| *Immobility* | 2.11 | 1.36 - 3.27 | **0.001** | - | - | - |
| Receiving cancer treatment | 0.43 | 0.17 - 1.06 | 0.07 | - | - | - |
| On oxygen therapy | 2.2 | 0.55 - 8.8 | 0.27 | - | - | - |
| - **Activity and participation** |  |  |  |  |  |  |
| Total Barthel Index score (basic ADLs) | 0.8 | 0.64 - 1.0 | 0.05 | 0.86 | 0.62 - 1.21 | 0.4 |
| Lawton Brody IADL score (instrumental ADLs) | 0.67 | 0.47 - 0.96 | 0.03 | 1.38 | 0.8 - 2.37 | 0.25 |
| WHODAS Summary score | 1.05 | 1.02 - 1.08 | **0.001** | - | - | - |
| *Cognition* | 1.15 | 0.96 - 1.36 | 0.12 | - | - | - |
| *Mobility* | 1.28 | 1.14 - 1.45 | **<0.001** | 1.41 | 1.14 - 1.74 | **0.002** |
| *Self-Care* | 1.22 | 1.02 - 1.46 | 0.03 | - | - | - |
| *Getting along with people* | 1.23 | 1 - 1.28 | 0.06 | - | - | - |
| *Household activities* | 1.18 | 1.03 - 1.31 | **0.01** | - | - | - |
| *Societal participation* | 1.11 | 1.03 - 1.21 | **0.009** | - | - | - |
| - **Personal Factors** |  |  |  |  |  |  |
| Age | 1.01 | 0.97 - 1.06 | 0.6 | 0.99 | 0.93 - 1.05 | 0.76 |
| Female | 1.27 | 0.51 - 3.13 | 0.61 | 0.98 | 0.29 - 3.25 | 0.96 |
| White British | 0.36 | 0.04 | 0.37 | - | - | - |
| Education above secondary school | 0.43 | 0.17 - 1.06 | 0.07 | - | - | - |
| CDSE: Confidence to receive help | 0.89 | 0.65 – 1.21 | 0.46 | - | - | - |
| - **Environmental factors** |  |  |  |  |  |  |
| Lives alone | 0.48 | 0.19 - 1.22 | 0.12 | 0.49 | 0.14 - 1.72 | 0.27 |
| Property with stairs | 0.52 | 0.18 – 1.51 | 0.23 | - | - | - |
| Months spent in physical and social isolation | 1.07 | 0.92 - 1.28 | 0.31 | - | - | - |
| Receiving community (or hospice) palliative care | 4.29 | 1.13 - 16.24 | 0.03 | - | - | - |
| Total number of ADL devices | 1.42 | 1.08 - 1.87 | **0.01** | 1.06 | 0.68 - 1.64 | 0.81 |
| Reduced physical activity indoors | 2.76 | 1.1 – 6.9 | 0.03 | - | - | - |
| Reduced physical activity outdoors, | 3.36 | 0.34 – 1.48 | **0.01** | 2.02 | 0.62 – 6.61 | 0.25 |
| **Constant (Increasing disability trajectory)** | **-** | **-** | **-** | **0.02** | **3.47e-06 – 148.07** | **0.4** |

NSCLC: non-small-cell lung cancer; ADL: activities of daily living; BADL: Basic activities of daily living; IADL: instrumental activities of daily living; WHODAS: World Health Organization disability assessment Schedule; CDSE: Chronic Disease Self-Efficacy subscale; Reduced physical activity includes responses of ‘little less’ or a ‘lot less’; Variables included in the multi-variable logistic regression were either continuous or dichotomous; Variables were selected for the multi-variable model if they had significance level of p≤0.01 in univariable logistic regression, were considered important factors in the systematic review, and did not show collinearity with other variables in the model.
